# Safety and efficacy of direct oral anticoagulants in comparison to warfarin in obese patients with atrial fibrillation: A systematic review and meta‐analysis

**DOI:** 10.1002/hsr2.2044

**Published:** 2024-04-21

**Authors:** Alla Adelkhanova, Prakash Raj Oli, Dhan Bahadur Shrestha, Jurgen Shtembari, Vivek Jha, Ghanshyam Shantha, George Michael Bodziock, Monodeep Biswas, Muhammad Omer Zaman, Nimesh K. Patel

**Affiliations:** ^1^ Department of Internal Medicine Mount Sinai Hospital Chicago Illinois USA; ^2^ Department of Internal Medicine Province Hospital Birendranagar Nepal; ^3^ Department of Internal Medicine John H. Stroger, Jr. Hospital of Cook County Chicago Illinois USA; ^4^ Department of Internal Medicine, Division of Cardiac Electrophysiology Wake Forest University School of Medicine Winston Salem North Carolina USA; ^5^ Department of Internal Medicine, Division of Cardiac Electrophysiology University of Maryland Medical Center Baltimore Maryland USA; ^6^ Department of Internal Medicine, Division of Cardiology Rudd Heart Hospital Louisville Kentucky USA; ^7^ Department of Cardiology Bon Secours Richmond Virginia USA

**Keywords:** anticoagulation, apixaban, atrial fibrillation, edoxaban, obesity, rivaroxaban

## Abstract

**Background and Aim:**

Obesity affects nearly 650 million adults worldwide, and the prevalence is steadily rising. This condition has significant adverse effects on cardiovascular health, increasing the risk of hypertension, coronary artery disease, heart failure, and atrial fibrillation (AF). While anticoagulation for obese patients with AF is a well‐established therapy for the prevention of thromboembolism, the safety and efficacy of different anticoagulants in this specific population are not well explored. This meta‐analysis aimed to compare direct oral anticoagulants (DOAC) to vitamin K antagonists in obese populations with AF.

**Methods:**

The PRISMA guidelines were followed for this meta‐analysis, registered in PROSPERO (CRD42023392711). PubMed, PubMed Central, Embase, Cochrane Library, and Scopus databases were searched for relevant articles from inception through January 2023. Two independent authors screened titles and abstracts, followed by a full‐text review in Covidence. Data were extracted in Microsoft Excel and analyzed using RevMan v5.4 using odds ratio as an effect measure.

**Results:**

Two thousand two hundred fifty‐nine studies were identified from the database search, and 18 were included in the analysis. There were statistically significant reductions in the odds of ischemic and hemorrhagic stroke in the DOAC group compared with the VKA group (OR 0.70, CI 0.66–0.75) and (OR 0.47, CI 0.35–0.62), respectively. In addition, the DOAC group exhibited lower odds of systemic embolism (OR 0.67, CI 0.54–0.83), major bleeding (OR 0.62, CI 0.54–0.72), and composite outcome (OR 0.72, CI 0.63–0.81).

**Conclusion:**

Based on the findings from this meta‐analysis, DOACs demonstrate superior safety and efficacy in obese patients with AF compared with VKAs. These results may have significant implications for guiding anticoagulation strategies in this patient population.

## 1 INTRODUCTION

Obesity is a body mass index (BMI) ≥30 kg/m^2^ among adults. It affects nearly 650 million adults worldwide, and its prevalence has almost tripled between 1975 and 2016.^1^ Obesity is known to have adverse effects on cardiovascular health, increasing the risk of hypertension, coronary artery disease, heart failure, and atrial fibrillation (AF).^2^ AF is the most common sustained cardiac arrhythmia and carries considerable morbidity and mortality.^3^ It has been established in the Framingham Heart Study that with every unit increase in BMI, the risk of AF increases by 4%–5%.^4^ Another meta‐analysis showed that there were 10%–29% greater increased risk of incident, postoperative, and postablative AF with every 5 unit increase in BMI.^5^ Given these implications, it is imperative to explore the consequences of AF in the obese population, including its complications and management.

Embolic stroke is the most dangerous complication of AF; therefore, its prevention is an essential consideration in AF management.^6^ Patients with AF are advised to start anticoagulation to lower the risk of embolic stroke, following a thorough discussion of the risks and benefits.^7^ Direct oral anticoagulants (DOACs) have been preferred over vitamin K antagonists (VKAs), such as warfarin, due to superior safety/efficacy, lack of required laboratory monitoring, fewer interactions with other drugs, and fewer dietary considerations.^8^ Both AHA/ACC/HRS (2023) and ESC (2020) recommended the benefits of DOACs over VKAs in OAC‐eligible AF patients. Still, they have not commented on the use of DOAC in AF patients with obesity, except AHA/ACC/HRS's recommendation of DOAC use among class III obesity patients with AF.^9^, ^10^

Obesity affects the pharmacokinetics of drugs by altering their volume of distribution (V_d_), peak concentration (C_max_), and drug exposure (area under curve, AUC), as well as drug clearance.^11^ Thus, obesity also affected the pharmacokinetics and pharmacodynamics of DOACs among obese patients.^12^ Due to concern about subanticoagulation with the use of fixed‐dose regimen, International Society on Thrombosis and Hemostasis (ISTH) (2016) recommended standard DOAC dosing for patients with a BMI ≤ 40 kg/m^2^ and weight ≤ 120 kg for prevention of ischemic stroke and systemic arterial embolism in nonvalvular AF while cautioning against DOAC use in patients with a BMI > 40 kg/m^2^ or weight > 120 kg due to limited data and potential pharmacokinetics or pharmacodynamic concerns. If DOACs need to be used in such patients, they are recommended to consider monitoring drug‐specific levels and, if below the expected range, consider switching to a VKA rather than adjusting the DOAC dose.^13^ Zhao et al. pointed out that obesity may have a modest effect on the pharmacokinetics of dabigatran, apixaban, rivaroxaban, or edoxaban. They highlighted that the standard doses of apixaban, rivaroxaban, and edoxaban are effective and safe in morbidly obese patients with AF. At the same time, the body weight is inversely affected by the peak concentration of dabigatran, with a significantly increased risk of gastrointestinal bleeding.^12^ There are now a growing number of studies studying the effectiveness and safety of the DOAC among obese or morbidly obese patients with AF, showing that they have better outcomes compared with those with normal BMI, and it's being depicted as an “obesity paradox.”^14^

Earlier meta‐analyses on the use of DOAC compared with warfarin in morbidly obese patients with AF showed mixed results.^15^, ^16^ However, these studies were unable to fully appraise the efficacy and safety of the DOAC compared with warfarin among obese as well as morbidly obese patients with AF. Therefore, this systematic review and meta‐analysis aimed to investigate the comparative safety and efficacy of DOACs compared with VKAs in obese patients with AF, defining safety as freedom from any major bleeding event and efficacy as freedom from stroke or systemic thromboembolism.

## 2 METHODS

### 2.1 Protocol

The PRISMA (Preferred Reporting Items for Systematic Reviews and Meta‐Analyses) guidelines were followed for this systematic review and meta‐analysis. The protocol is registered in PROSPERO 2023 CRD42023392711. The PRISMA checklist is included in a supplementary file (Supplementary material).

### 2.2 Search strategy

PubMed, PubMed Central, Embase, Cochrane Library, and Scopus databases were searched in January 2023. An appropriate combination of search words such as “atrial fibrillation,” “direct oral anticoagulant,” “DOAC,” “vitamin K antagonist,” “Warfarin,” “obesity” and applicable Boolean operators were used. The search method will be described in detail in a supplemental file.

### 2.3 Eligibility criteria

This meta‐analysis contained prospective and retrospective studies in which obese patients with nonvalvular AF received either DOAC or Warfarin and included case‐control, cohort, and randomized control trials (RCTs). Conference abstracts, editorials, comments, qualitative and viewpoint articles, case reports, review articles, and other meta‐analyses were excluded.

### 2.4 Outcomes measured

The primary efficacy outcome was a composite of stroke, systemic embolism, myocardial infarction (MI), or any cause of death. The secondary outcomes were ischemic stroke, systemic embolism, and all‐cause mortality. The primary safety outcome was major bleeding. We also analyzed the outcome of all‐cause mortality.

### 2.5 Study selection

Two independent reviewers screened the titles and abstracts using Covidence, while a third reviewer resolved conflicts. Two reviewers completed the full‐text review, and conflicts were resolved by another reviewer among the list of authors. Data was then extracted for qualitative and quantitative processing.

### 2.6 Data extraction

A standardized form was designed in Microsoft Excel to extract pertinent data, including study authors, study details, quality, and endpoints. The endpoints of this meta‐analysis were all‐cause mortality, ischemic stroke, systemic embolism, a composite of ischemic stroke and systemic embolism, and a major bleeding event.

### 2.7 Study quality

The quality of individual articles was assessed using the Joanna Briggs Institute's critical appraisal (JBI) tools for the risk of bias^17^ (Supporting Information: Table 1). ROB‐2 tool used for risk of bias assessment of RCTs^18^ (Supporting Information: eFigure 1). Two authors independently assessed each study design and the number of patients with each outcome. A third person then resolved conflicts.

### 2.8 Data analysis

Data was analyzed using RevMan v5.4.^19^ An odds ratio (OR) was used for outcomes such as mortality, ischemic stroke, systemic embolism, composite of ischemic stroke and systemic embolism, and a major bleeding event.

Heterogeneity was measured by the *I* ^2^ test among the included studies. A random effect model was used for analysis to consider heterogeneity.

Sensitivity analysis was performed based on the type of DOAC used and BMI class to test the robustness of the analysis.

## 3 RESULTS

**Figure 1:**
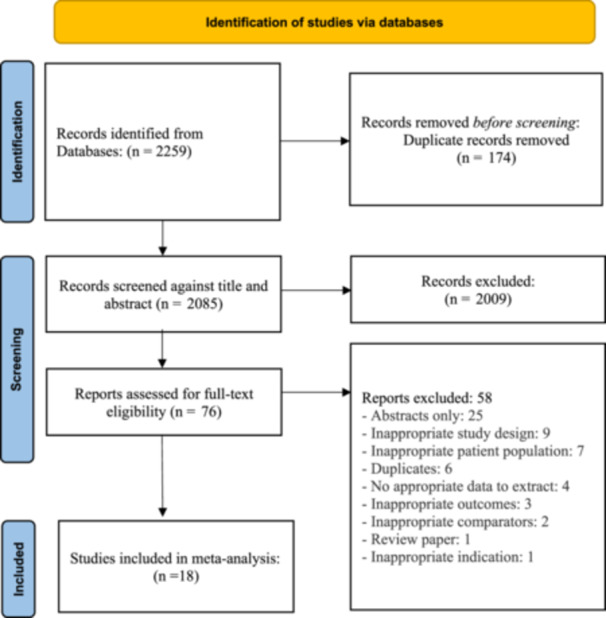
PRISMA 2020 flow diagram for the systematic review.

Among 2259 studies identified from the database search, 2085 were screened for title and abstract after removing 174 duplicates. After excluding 2009 studies during title and abstract screening, full text of 76 studies were assessed for eligibility. Fifty‐eight studies were excluded from the full‐text review, and 18 were included in the analysis. Among the 18 studies included, 16 were retrospective cohort studies, and 2 were randomized controlled trials. The PRISMA flow diagram for the review is shown in Figure 1.

### 3.1 Qualitative analysis

Eighteen studies involving 387,205 obese patients with AF were included in this meta‐analysis. Among 387,205 patients, 193,947 (50.09%) patients received DOAC whereas 193,258 (49.91%) patients received warfarin. Among 193,947 patients who received DOAC, 130,634 (67.36%) patients received rivaroxaban, 41,540 (21.42%) patients received apixaban, 13,063 (6.74%) patients received dabigatran, 6234 (3.21%) patients received edoxaban, and 2476 (1.28%) patients received unspecified DOAC agent. Among 386,071 patients with gender data, 249,813 (64.71%) were male while 136,258 (35.29%) were female. The average mean age was 69.16 ± 9.80 years. The baseline patient characteristics, underlying comorbidities, clinical parameters, baseline medications, and clinical outcomes were collected and analyzed, as presented in Tables 1 and 2 and Supporting Information: Table 2.

**Table 1:** Baseline characteristics of studies and participants, including their comorbidities.

Study	Publication year	Country	Study design	No. of patients		Intervention	Age (years), mean (SD)	Gender	BMI, %			Comorbidities, %							
								Male, %	30.0–34.9 kg/m^2^	35.0–39.9 kg/m^2^	≥40.0 kg/m^2^	Hypertension	Hyperlipidemia	Diabetes mellitus	COPD	Congestive heart failure	Coronary artery disease	Cerebrovascular disease	Peripheral vascular disease
Alberts et al.^20^	2022	USA	Retrospective cohort study	*N* = 95,875	Rivaroxaban *n* = 33,191	Rivaroxaban	62.97 (10.3)	65.9	49.2	15.5	35.2	85.5	64.5	53.89	13.5	22.3	16.2	11.9	12.5
					Warfarin *n* = 62,684	Warfarin	67.72 (10.3)	62.4	52.4	15.0	32.6	81.6	60.6	70.54	19.4	34.5	16.2	18.1	18.0
Berger et al.^21^	2021	USA	Retrospective cohort study	*N* = 15,635	Rivaroxaban *N* = 10,555	Rivaroxaban	59.3 (8.6)	69			37.7	86.6	67.9	42.5	10.1	34.0	33.4	11.15	8.1
					Warfarin *N* = 5080	Warfarin	59.4 (8.9)	68.1			39.0	87.1	67.9	43.1	10.2	34.0	33.4	13.76	7.6
Boivin‐Proulx et al.^22^	2022	Canada	Retrospective cohort study	N = 2195	Rivaroxaban, *n* = 403	Rivaroxaban 20 mg once daily	71.91 (8.09)	45.57				88.06	62.09	60.72	49.63	33.52	55.48		22.57
					Apixaban *n* = 539	Apixaban 5 mg twice daily	74.22 (8.26)	44.45					61.99	62.26	47.24	43.71	56.51		20.0
					Warfarin *n* = 1253	Warfarin	72.83 (11.07)	43.71				87.28	61.20	59.93	46.58	43.12	56.35		20.85
Boriani et al.^23^	2018	46 countries	Three‐group, randomized, double‐blind, double‐dummy study	*N* = 8457	Higher‐dose edoxaban *n* = 2876	Edoxaban 60 mg dose daily	69.96 (10.52)	59.60	61.34	24.24	14.42	97.32		47.64		62.83		22.25	
					lower‐dose edoxaban *n* = 2828	Edoxaban 30 mg dose daily	69.59 (10.31)	58.10	61.60	25.32	13.08	97.21		46.92		60.08		22.70	
					Warfarin *n* = 2753	Warfarin	68.46 (10.44)	60.04	61.86	24.92	13.22	97.78		47.69		61.24		21.58	
Briasoulis et al.^24^	2021	USA	Retrospective cohort study	*N* = 28,011	Apixaban (*n* = 6052)	Apixaban 5 mg or 2.5 mg twice daily	69.9	99				84.9		22		31.1	26.8	7	11.4
					Dabigatran (*n* = 4233)	Dabigatran 150 mg twice daily	65.7	99				84.5		29.1		26.2	24.8	5	8.4
					Rivaroxaban (*n* = 4309)	Rivaroxaban 20 mg or 15 mg once daily	66.7	99				83.2		25.9		27.7	24.6	4.5	8.8
					Warfarin (*n* = 13,417)	Warfarin	66.5	98.9				86.8		31.8		35.8	29	7.3	12
Costa et al.^25^	2020	USA	Retrospective cohort study	*N* = 71,226	Rivaroxaban *n* = 35,613	Rivaroxaban	67.35 (11.12)	60.5	48.0	26.7	25.2	78.9		33.7	13.5	13.5		4.7	8.4
					Warfarin *n* = 35,613	Warfarin	68.3 (10.38)	59.8	47.9	26.7	25.4	78.9		35.4	14.5	14.0		5.3	9.0
Deitelzweig et al.^26^	2020	USA	Retrospective cohort study	*N* = 88,461	Apixaban *n* = 21,242	Apixaban	71.5 (9.9)	51.9				94.3		53.6		38.0			
					Dabigatran *n* = 7171	Dabigatran	69.6 (10.0)	56.2				93.0		52.7		34.7			
					Rivaroxaban *n* = 29,146	Rivaroxaban	70.0 (10.3)	53.7				93.2		52.0		35.2			
					Warfarin *n* = 30,902	Warfarin	72.8 (8.8)	51.7				95.1		61.4		47.6			
Deitelzweig et al.^27^	2022	USA	Retrospective cohort study	*N* = 26,522	Apixaban *n* = 13,604	Apixaban 5 mg or 2.5 mg twice daily	75.4 (7.6)	99				87		51		32	54	9	22
					Warfarin *n* = 12,918	Warfarin	74.4 (7.9)	99				87		56		37	53	10	22
Huang et al.^28^	2021	USA	Retrospective cohort study	*N* = 3318	Dabigatran (*n* = 1659)	Dabigatran	66.41 (9.13)	64	5.1	23.4	71.5	68.6		54.2		47.1		4.5	35.3
					Warfarin (*n* = 1659)	Warfarin	66.43 (10.31)	62.6	4.9	23.2	71.3	69.5		54.2		47		4.6	34.9
Kido and Ngorsuraches^29^	2019	USA	Retrospective cohort study	*N* = 128	DOAC (*n* = 64)	Apixaban, dabigatran, and rivaroxaban	64.28 (10.16)	60.94										18.75	
					Warfarin (*n* = 64)	Warfarin	65.88 (12.18)	54.69										15.62	
Kushnir et al.^30^	2019	USA	Retrospective cohort study	*N* = 429	Apixaban (*n* = 103)	Apixaban	65·9 (10·7)	44			100								
					Rivaroxaban (*n* = 174)	Rivaroxaban	60·9 (12·6)	45			100								
					Warfarin (*n* = 152)	Warfarin	66·8 (13·6)	41			100								
Lip et al.^31^	2019		Multicenter, prospective, randomized, open, blinded endpoint trial	*N* = 1067	Edoxaban (*n* = 530)	Edoxaban 60 mg daily	62.9 (9.3)					84.5		25.8		48.3	19.2	4.9	2.8
					Enoxaparin– Warfarin (*n* = 537)	Warfarin	63.2 (10.1)					86.4		25.9		45.6	19.4	5.0	4.5
Nakao et al.^32^	2022	UK	Retrospective cohort study	*N* = 4066	DOACs *n* = 2033	DOACs	74.83 (9.18)	53.91	61.44	38.56		89.87		43.53	21.94	21.50	15.59	17.81	7.97
					Warfarin *n* = 2033	Warfarin	74.95 (8.53)	55.14	61.44	38.56		89.72		43.48	22.28	22.04	15.74	18.35	7.33
Patil and Lebrecht^33^	2020	USA	Retrospective cohort study	*N* = 236	DOAC (*n* = 129)	Dabigatran 75/150 mg twice daily, rivaroxaban 15/20 mg daily and apixaban 2.5/5 mg twice daily	70.46 (7.05)	99.22				91.47		68.22		28.68		9.30	
					Warfarin (*n* = 107)	Warfarin	70.52 (6.31)	97.20				92.52		59.81		37.38		8.41	
Perales et al.^34^	2020	USA	Retrospective cohort study	*N* = 67	Rivaroxaban (*n* = 37)	Rivaroxaban													
					Warfarin (*n* = 30)	Warfarin													
Peterson et al.^35^	2019	USA	Retrospective cohort study	*N* = 9474	Rivaroxaban (*n* = 4543)	Rivaroxaban	61.8 (10.8)	55.0				87.2	61.1	47.7		30.8			13.6
					Warfarin (*n* = 4931)	Warfarin	64.4 (10.8)	52.8				88.2	63.0	57.6		45.0			21.1
Russo et al.^36^	2020	Italy	Retrospective cohort study	*N* = 960	DOACs (*n* = 250)	Dabigatra 110/150 mg twice daily, rivaroxaban 20 mg daily, edoxaban 60 mg daily, and apixaban 5 mg twice daily	66.5 (9.1)	48.8				48.8		14.8		20	16	6	
					Warfarin (*n* = 710)	Warfarin	68.8 (10.4)	48.1				49.01		13.9		20.9	15.9	5.2	
Weir et al.^37^	2021	USA	Retrospective cohort study	*N* = 31,078	Rivaroxaban (*n* = 12,663)	Rivaroxaban	68.9 (9.5)	60.0	40.3	15.3	44.4	95.8	85.8		25.1	37.0	33.2	15.5	15.3
					Warfarin (*n* = 18,415)	Warfarin	70.8 (8.5)	57.9	44.2	13.6	42.2	96.1	85.2		31.2	51.2	34.8	21.5	21.0

**Table 2:** Clinical efficacy and safety outcomes among the included participants.

Study	Groups	Composite of stroke, systemic embolic event, major bleeding, or death				Ischemic stroke				Hemorrhagic stroke	Systemic embolism				Major bleeding				Intracranial bleeding	GI bleeding	All‐cause mortality
		Total	30.0–34.9 kg/m^2^	35.0–39.9 kg/m^2^	≥40.0 kg/m^2^	Total	30.0–34.9 kg/m^2^	35.0–39.9 kg/m^2^	≥40.0 kg/m^2^		Total	30.0–34.9 kg/m^2^	35.0–39.9 kg/m^2^	≥40.0 kg/m^2^	Total	30.0–34.9 kg/m^2^	35.0–39.9 kg/m^2^	≥40.0 kg/m^2^			
Alberts et al.^20^	Rivaroxaban	926/21,442	473/10,755	121/3040	332/7647	742/21,442	374/10,755	99/3040	269/7647	194/21,442	72/21,442	34/10,755	11/3040	27/7647	421/21,442	223/10,755	53/3040	145/7647			
	Warfarin	1199/21,442	620/10,755	185/3040	394/7647	936/21,442	479/10,755	137/3040	320/7647	311/21,442	110/21,442	55/10,755	20/3040	35/7647	422/21,442	200/10,755	54/3040	168/7647			
Berger et al.^21^	Rivaroxaban	366/10,555			366/10,555	186/10,555				46/10,555	26/10,555				366/3958			288/3792			
	Warfarin	222/5080			222/5080	106/5080				39/5080	14/5080				312/2604			230/2094			
Boivin‐Proulx et al.^22^	Rivaroxaban	43/403				3/403				0/403	5/403				9/403				1/403	0/403	20/403
	Apixaban	41/539				3/539				0/539	1/539				6/539				0/539	2/539	24/539
	Warfarin	96/1253				9/1253				2/1253	2/1253				33/1253				5/1253	15/1253	70/1253
Boriani, G. et al.^23^	Higher dose edoxaban	508/2876	318/1764	117/697	73/415										185/2876	119/1764	36/697	30/415			284/2876
	Lower dose edoxaban	445/2828	285/1742	112/716	48/370										122/28,282	69/1742	40/716	15/370			244/28,282
	Warfarin	498/2753	324/1703	114/686	60/364										214/2753	130/1703	54/686	28/364			265/2753
Briasoulis et al.^24^	Apixaban					32/6052				7/6052					99/6052					68/6052	328/6052
	Dabigatran					29/4233				2/4233					64/4233					50/4233	183/4233
	Rivaroxaban					26/4309				7/4309					91/4309					59/4309	177/4309
	Warfarin					124/13,417				53/13,417					583/13,417					381/13,417	1047/13,417
Costa et al.^25^	Rivaroxaban	429/35,613	212/16,821	115/9428	105/9161	399/35,613	196/16,821	106/9428	100/9161						877/35,613	420/16,821	231/9428	226/9161	79/35,613		
	Warfarin	668/35,613	343/16,821	163/9428	157/9161	586/35,613	307/16,821	142/9428	137/9161						1382/35,613	630/16,821	352/9428	392/9161	164/35,613		
Deitelzweig et al.^26^	Apixaban	132/21,242				107/21,242				23/21,242					399/21,242				38/21,242	195/21,242	
	Dabigatran	67/7171				56/7171									174/7171				17/7171	110/7171	
	Rivaroxaban	226/29,146				170/29,146				41/29,146	17/29,146				1050/29,146				67/29,146	612/29,146	
	Warfarin	406/30,902				276/30,902				115/30,902	20/30,902				1491/30,902				190/30,902	721/30,902	
Deitelzweig et al.^27^	Apixaban	147/13,604				109/13,604				29/13,604	11/13,604				398/13,604				68/13,604	210/13,604	
	Warfarin	218/12,918				148/12,918				56/12,918	17/12,918				779/12,918				163/12,918	384/12,918	
Huang et al.^28^	Dabigatran	118/1659				118/1659					10/1659								37/1659	329/1659	142/1659
	Warfarin	224/1659				224/1659					13/1659								77/1659	395/1659	570/1659
Kido and Ngorsuraches^29^	DOAC					4/64									5/64						
	Warfarin					3/64									12/64						
Kushnir et al.^30^	Apixaban					1/103									3/103						
	Rivaroxaban					4/174									5/174						
	Warfarin					2/152									12/152						
Lip et al.^31^	Edoxaban	4/530													2/517						
	Warfarin	5/537													4/528						
Nakao et al.^32^	DOAC					51/2033	38/1249								70/2033	47/1249					
	Warfarin					67/2033	42/1249								99/2033	63/1249					
Patil et al.^33^	DOAC	3/129													7/129						
	Warfarin	5/107													9/107						
Perales et al.^34^	Rivaroxaban														4/37						0/37
	Warfarin														0/30						3/30
Peterson et al.^35^	Rivaroxaban	52/3563			52/3563										77/3563			77/3563			
	Warfarin	59/3563			59/3563										96/3563			96/3563			
Russo et al.^36^	DOAC										5/248				8/248						1/248
	Warfarin										19/496				34/496						3/496
Weir et al.^37^	Rivaroxaban	272/9999	120/4086	40/1485	112/4344	216/9999	94/4086	35/1485	87/4344	59/9999	19/9999	9/4086	2/1485	8/4344	262/9999	103/4086	41/1485	123/4344			
	Warfarin	396/9999	168/4086	54/1485	152/4344	322/9999	129/4086	39/1485	119/4344	93/9999	29/9999	18/4086	8/1485	13/4344	285/9999	116/4086	39/1485	124/4344			

### 3.2 Quantitative analysis

#### 3.2.1 Composite outcome

**Figure 2:**
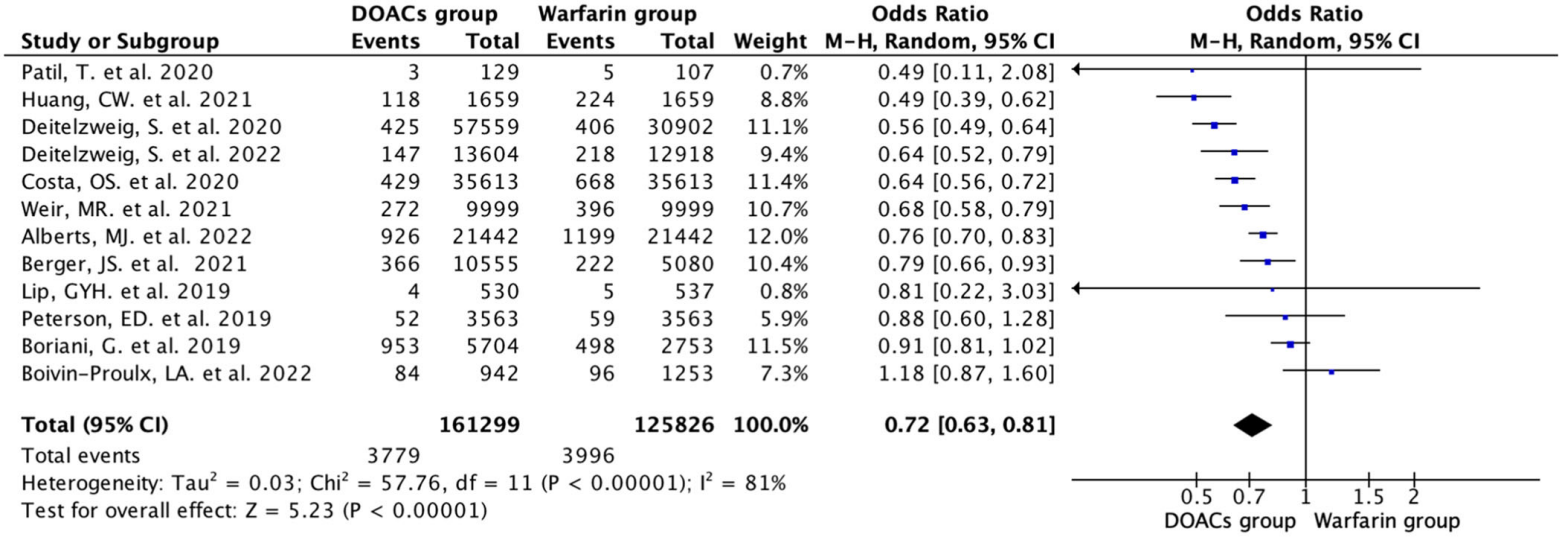
Forest plots show a significantly lower occurrence of composite events in the DOAC group than in the Warfarin group using the random effect model.

Twelve studies reported the composite events with an incidence rate of 2.71% (*N* = 7775/287,125) [DOAC group (2.34%; *N* = 3779/161,299) vs. Warfarin group (3.17%; *N* = 3996/125,826)]. Pooled data analysis showed a 28% lower occurrence of the composite events in the DOAC group compared with the Warfarin group (OR 0.72, 95% CI 0.63–0.81; *n* = 287,125; *I* ^2^ = 81%) (Figure 2). In the subanalysis comparing the specific DOACs to warfarin, the composite events had significantly lower occurrence in rivaroxaban (OR 0.74, 95% CI 0.65–0.85), apixaban (OR 0.65, 95% CI 0.45–0.93), and dabigatran (OR 0.59, 95% CI 0.41–0.84), but not so for the edoxaban subgroup despite favoring it (OR 0.91, 95% CI 0.81–1.02), (Supporting Information: eFigure 2).

#### 3.2.2 Stroke

**Figure 3:**
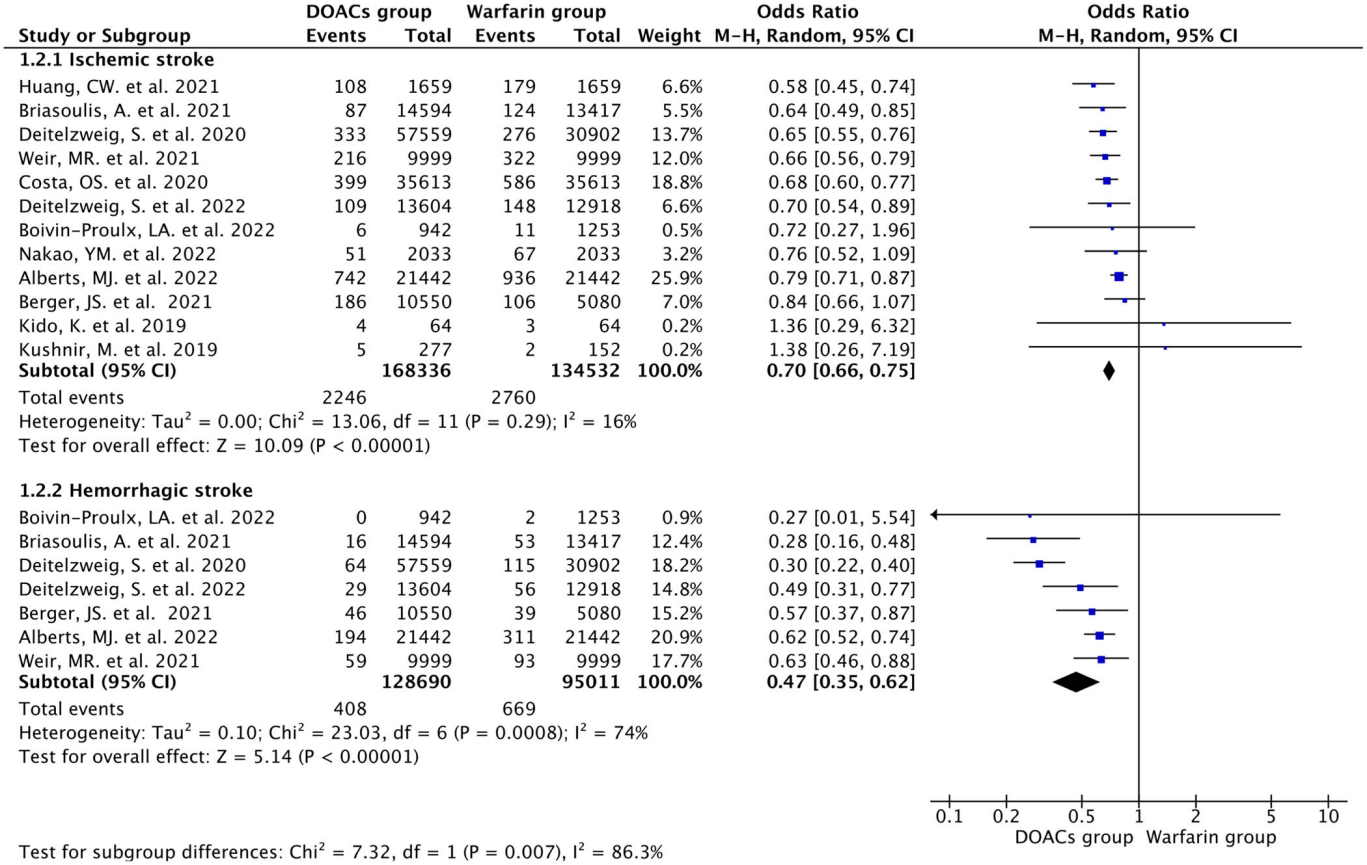
Forest plot showing significantly lower occurrence of stroke events in the DOAC group compared with Warfarin group using random effect model.

Twelve studies reported the ischemic stroke (IS) events with an incidence rate of 1.65% (*N* = 5006/302,868) [DOAC group (1.33%; *N* = 2246/168,336) vs. Warfarin group (2.05%; *N* = 2760/134,532)]. Pooled data analysis showed a 30% lower occurrence of IS events in the DOAC group compared with Warfarin group (OR 0.70, 95% CI 0.66–0.75; *n* = 302,868; *I* ^2^ = 16%). Seven studies reported hemorrhagic stroke events with and incidence rate of 0.48% (*N* = 1077/223,701) [DOAC group (0.32%; *N* = 408/128,690) vs. Warfarin group (0.70%; *N* = 669/95,011)] and pooled data showed a 53% lower occurrence of the hemorrhagic stroke in the DOAC group compared with the Warfarin group (OR 0.47, 95% CI 0.35–0.62; *n* = 223,701; *I* ^2^ = 74%) (Figure 3).

In subanalysis comparing different DOAC agents with the warfarin group, the occurrences of ischemic stroke as well as hemorrhagic stroke were significantly lower in the rivaroxaban subgroup (ischemic stroke: OR 0.72, 95%CI 0.66–0.78 and hemorrhagic stroke: OR 0.55; 95% CI 0.45–0.66), apixaban subgroup (ischemic stroke: OR 0.61; 95% CI 0.52–0.71 and hemorrhagic stroke: OR 0.36; 95% CI 0.27–0.49), and dabigatran subgroup (ischemic stroke: OR 0.71; 95%CI 0.54–0.93 and hemorrhagic stroke: OR 0.12, 95% CI 0.03–0.49)] (Supporting Information: eFigure 3a,b).

#### 3.2.3 Systemic embolic events

**Figure 4:**
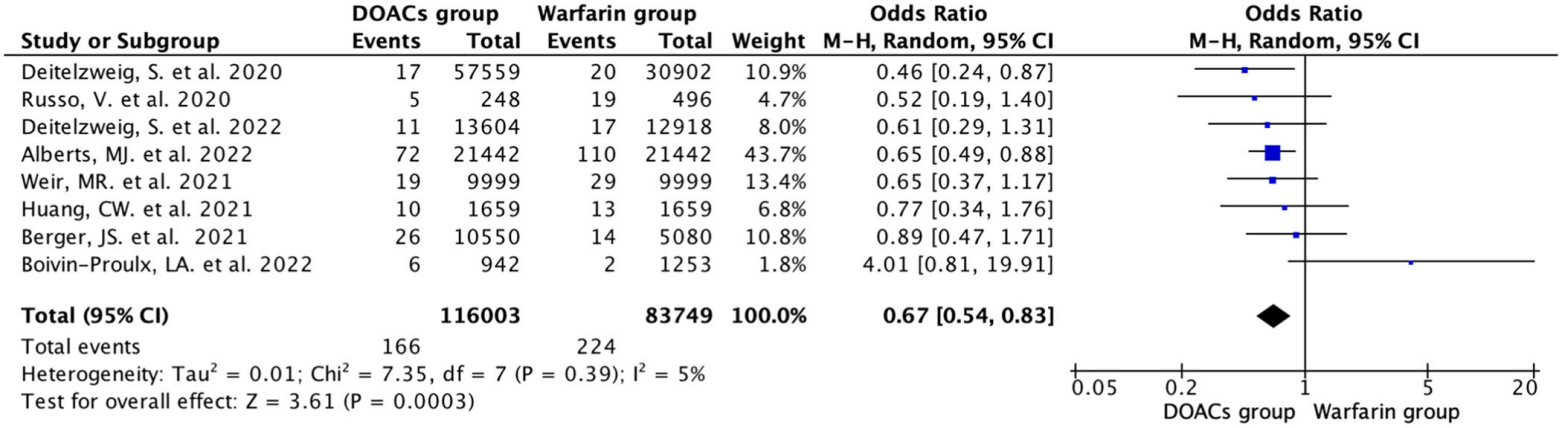
Forest plot showing significantly lower occurrence of systemic embolic events in the DOAC group compared with the Warfarin group using the random effect model.

Eight studies reported systemic embolism events with an incidence rate of 0.20% (*N* = 390/199,752) (DOAC group: 0.14%; *N* = 166/116,003 vs. Warfarin group: 0.27%; *N* = 224/83,749). Pooled data analysis showed a 33% lower occurrence of systemic embolism in the DOAC group compared with the warfarin group (OR 0.67, 95% CI 0.54–0.83; *n* = 199752; *I* ^2^ = 5%) (Figure 4). In the subanalysis comparing different DOAC agents with the warfarin group, there were no significant difference occurrence of systemic embolic events for three DOAC agents: rivaroxaban, apixaban, and dabigatran (Supporting Information: eFigure 4).

#### 3.2.4 Major bleeding

**Figure 5:**
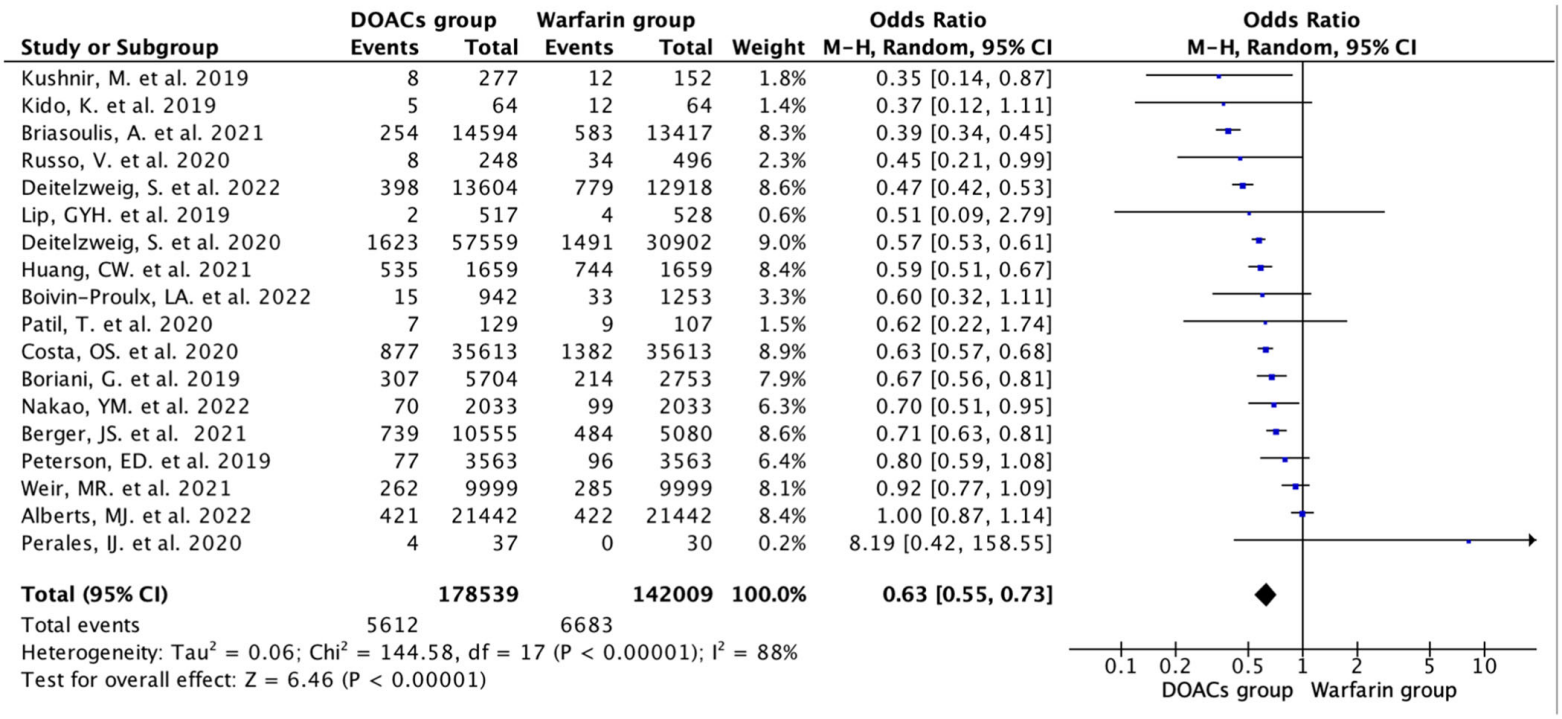
Forest plot showing significantly lower occurrence of major bleeding events in the DOAC group compared with VKA group using random effect model.

Eighteen studies reported major bleeding events with an incidence rate of 3.84% (*N* = 12,295/320,548) [DOAC group: 3.14%; *N* = 5612/178,539 vs. Warfarin group: 4.7%; *N* = 6683/142,009). Pooled data showed a 37% lower occurrence of the major bleeding events in DOAC group compared with warfarin group (OR 0. 63, 95% CI 0.55–0.73; *n* = 320,548; *I* ^2^ = 88%) (Figure 5). Among different bleeding event types, the DOAC group had significantly lower occurrences of these bleeding types compared with the warfarin group [Intracranial Hemorrhage (ICH): OR 0.40, 95% CI 0.35–0.46; *n* = 192,466; *I* ^2^ = 0% and Gastrointestinal (GI) bleeding: OR 0.57, 95% CI 0.44–0.73; *n* = 148,507; *I* ^2^ = 89%] (Supporting Information: eFigure 5).

In the subanalysis comparing different DOAC agents with the warfarin group, there were significantly lower occurrences of the major bleedings in rivaroxaban (OR 0.73, 95% CI 0.63–0.85) apixaban (OR 0.41, 95% CI 0.35–0.47) and edoxaban (OR 0.67, 95% CI 0.56–0.81) subgroups, but not in the dabigatran (OR 0.91, 95% CI 0.51–1.62) subgroup (Supporting Information: eFigure 6). In the subanalysis comparing different DOAC agents with the warfarin group, there was a significantly lower occurrences of GI bleeding in the rivaroxaban (OR 0.58, 95% CI 0.37–0.91) apixaban (OR 0.39, 95% CI 0.34–0.44) and dabigatran (OR 0.61, 95% CI 0.43–0.85) subgroups (Supporting Information: eFigure 7).

In the subanalysis comparing different DOAC agents with the warfarin group, there was significantly lower occurrences of intracranial hemorrhages in the rivaroxaban (OR 0.43, 95% CI 0.35–0.52) apixaban (OR 0.35, 95% CI 0.28–0.43) and dabigatran (OR 0.43, 95% CI 0.32–0.59) subgroups (Supporting Information: eFigure 8).

#### 3.2.5 All‐cause mortality

Seven studies reported the all‐cause mortality events, and the pooled data showed a significantly lower occurrence of all‐cause mortality in the DOAC group by 44% compared with the warfarin group (OR 0.56, 95% CI 0.34–0.94; *n* = 46,858; *I* ^2^ = 97%) (Supporting Information: eFigure 9). In the subanalysis comparing the different DOAC agents with the warfarin group, there was a significant reductions of the all‐cause mortality in the rivaroxaban (OR 0.65, 95% CI 0.46–0.91) and apixaban (OR 0.66, 95% CI 0.48–0.91) subgroups but not in dabigatran (OR 0.32, 95% CI 0.10–1.04) and edoxaban (OR 0.96, 95% CI 0.82–1.12) subgroups (Supporting Information: eFigure 10).

#### 3.2.6 Subanalysis based on BMI classes

Subanalysis of the clinical efficacy and safety of DOAC agents compared with warfarin use was performed based on the obesity classification: obesity class I (30–34.9 kg/m^2^), obesity class II (35–39.9 kg/m^2^), and obesity class III (>40.0 kg/m^2^). We found that the use of DOACs was associated with statistically significant reductions in the composite outcome of ischemic stroke, systemic embolism, and major bleeding across all three obesity classes. However, the individual outcomes of systemic embolism in obesity classes I and III and the major bleeding in obesity classes I and II were not significant.

#### 3.2.7 Composite outcome

In the subanalysis across different obesity classes, there was a significantly lower occurrence of the composite outcomes in all three obesity classes: obesity class I (OR 0.74, 95% CI 0.62–0.90), obesity class II (OR 0.76, 95% CI 0.59–0.97) and obesity class III (OR 0.72, 95% CI 0.60–0.87) in comparison to the warfarin group (Supporting Information: eFigure 11).

#### 3.2.8 Ischemic stroke

In the subanalysis across different obesity classes, there was a significantly lower occurrence of ischemic stroke in all three obesity classes: obesity class I (OR 0.73, 95% CI 0.64–0.82), class II (OR 0.75, 95% CI 0.63–0.89), and class III (OR 0.73, 95% CI 0.63–0.85) on comparison with warfarin group (Supporting Information: eFigure 12).

#### 3.2.9 Systemic embolism

In the subanalysis across different obesity classes, there was a significantly lower occurrence of systemic embolic events only in obesity class II (OR 0.47, 95% CI 0.24–0.92) but not in obesity class I (OR 0.81, 95% CI 0.37–1.79) of class III (OR 0.73, 95% CI 0.47–1.13), in comparison to warfarin group (Supporting Information: eFigure 13).

#### 3.2.10 Major bleeding

In the subanalysis across different obesity classes, there was a significantly lower occurrence of systemic embolic events only in obesity class III (OR 0.75, 95% CI 0.62–0.90), however not in obesity class I (OR 0.80, 95% CI 0.64–1.01) or class II (OR 0.78, 95% CI 0.61–1.00), in comparison to warfarin group (Supporting Information: eFigure 14).

#### 3.2.11 Publication bias

Publication bias for the composite outcome, stroke, major bleeding, and all‐cause mortality was checked with a Funnel plot, which showed the asymmetric distribution of studies signifying significant publication bias (Supporting Information: eFigure 15).

## 4 DISCUSSION

Obesity is a well‐established risk factor for AF, which itself carries a high risk of major life‐threatening thromboembolism and ischemic stroke.^38^ Thus, primary as well as secondary prevention of the thromboembolism and ischemic stroke risk with anticoagulation is one of the cornerstones of AF management in suitable AF patients.^39^ Due to the better clinical efficacy profile (systemic embolism and the stroke) as well as the clinical safety (major bleeding and intracranial hemorrhage), thus higher mortality benefit, of DOACs over the warfarin, DOACs are recommended over warfarin for the anticoagulation in AF patients in the 2023 ACC/AHA/ACCP/HRS guideline. However, there was little clinical evidence to support this clinical safety and efficacy superiority profiles of DOACs over warfarin among obese patients with AF. So, 2023 ACC/AHA/ACCP/HRS guideline recommends DOAC among AF patients with class III obesity (class of recommendation 2a and the level of evidence B‐NR) only while no comments regarding which type of anticoagulants is suitable for AF patients with class I or II obesity.^10^ Therefore, it is imperative to investigate the safety and efficacy of anticoagulation in AF patients with obesity. This comprehensive systematic review and meta‐analysis evaluated the efficacy and safety of DOACs, as compared with VKAs, within the obese patient population suffering from nonvalvular AF.

Our meta‐analysis revealed that obese patients with AF who received DOACs, as compared with VKAs, had significantly lower occurrences of composite events as well as individual events: stroke (ischemic as well as hemorrhagic) and systemic embolic events, in overall. The DOACs also significantly lowered major bleeding rates, including GI bleeding, ICH, and all‐cause mortality in this patient cohort. Among different DOAC agents, rivaroxaban and apixaban use had significantly lower occurrence of composite events, ischemic as well as hemorrhagic strokes, major bleeding including GI bleeding as well as ICH, and all‐cause mortality compared with warfarin use. Dabigatran use had a significantly lower occurrence of composite events, GI bleeding, and ICH than warfarin use. Across all three classes of obesity, the DOAC had significantly lower occurrences of composite events as well as ischemic stroke events. Whereas only class II obesity and class III obesity had a significantly lower occurrence of systemic embolism events and major bleeding, respectively, when using DOACs compared with warfarin. None of the DOAC agents were associated with a significant reduction of systemic embolic events on individual comparison with warfarin use. Similar findings were reported on this topic in the previous other studies.

A real‐world electronic health record study by Costa et al. demonstrated a significant reduction in stroke and systemic embolism, along with a reduction in major bleeding, with rivaroxaban in comparison to warfarin use in obese patients with AF.^40^ In this study, there were no significant reductions in stroke and systemic embolism, and major bleeding events across different BMI classes. In contrast, in our study, there was a statistically significant reduction in both systemic embolism and major bleeding across obesity classes in the DOAC group, except the systemic embolism in obesity classes I and III, and the major bleeding in obesity classes I and II, where reduction was not statistically significant. These disparities in our findings and by Costa et al. among different BMI classes seem to be due to the type of DOACs used, differences in the number of patients in different BMI classes, and differences in the statistical analysis used.

The post‐hoc analysis of the ARISTOTLE trial based on the obesity performed by Deitelzweig et al. showed a lower risk of stroke and systemic embolism in apixaban and rivaroxaban groups compared with the warfarin group; however, the dabigatran group had similar rates of stroke and systemic embolism as the warfarin group, while all three DOACs were associated with lower major bleeding rates than warfarin.^26^ These findings contrast with our subanalysis, which showed that compared with warfarin, apixaban, rivaroxaban, and dabigatran all have significantly lower stroke rates; however, major bleeding rates were only significantly lower in apixaban and rivaroxaban groups. One potential explanation for the discrepancy in outcomes could be the mechanism of action of DOACs, as dabigatran is a factor IIa inhibitor while apixaban and rivaroxaban are factor Xa inhibitors.^41^ The pharmacokinetics of dabigatran also differ from apixaban and rivaroxaban since dabigatran undergoes hepatic glucuronidation, while apixaban and rivaroxaban are metabolized through the cytochrome P450 system.^41^

A retrospective study by Briasoulis et al. interestingly reported that in patients weighing over 120 kg, apixaban had a higher risk of stroke than warfarin, while rivaroxaban and dabigatran had a similar risk as warfarin, and all three DOACs had a lower bleeding risk.^24^ This differs from the results of our study and may be partially explained by the diversity in comorbidity burden among the various DOACs and the differences in the patient population.^42^

Our results do have some limitations. First, we did not make a comparison of obese to nonobese or underweight populations. Second, the data set did not include INR levels in patients on warfarin, and it's possible that subtherapeutic or supratherapeutic warfarin effects could influence the rates of stroke and bleeding. Despite these limitations, the meta‐analysis has multiple strengths, including a large number of studies and a large patient population, increasing the power of the results. The analysis also compared different individual DOACs to warfarin and allowed subanalysis of various obesity classes.

## 5 CONCLUSION

DOACs appear to show superior safety and efficacy (stroke, systemic embolism, MI, bleeding, or death) when compared with VKAs (warfarin) in obese populations with AF. As the totality of this evidence mostly came from observational studies, additional data from larger randomized controlled trials will be required to discern the appropriate DOACs, dosage regimens, and BMI extremes.

## AUTHOR CONTRIBUTIONS

**Alla Adelkhanova**: Conceptualization; data curation; methodology; project administration; resources; software; writing—original draft; writing—review and editing. **Prakash Raj Oli**: Data curation; formal analysis; methodology; project administration; resources; software; validation; writing—original draft; writing—review and editing. **Dhan Bahadur Shrestha**: Conceptualization; data curation; formal analysis; methodology; project administration; resources; software; validation; writing—original draft; writing—review and editing. **Jurgen Shtembari**: Data curation; methodology; project administration; resources; software; writing—original draft; writing—review and editing. **Vivek Jha**: Data curation; methodology; resources; software; writing—original draft; writing—review and editing. **Ghanshyam Shantha**: Methodology; project administration; supervision; writing—review and editing. **George Michael Bodziock**: Investigation; project administration; supervision; validation; writing—review and editing. **Monodeep Biswas**: Methodology; project administration; supervision; validation; writing—review & editing. **Muhammad Omer Zaman**: Investigation; project administration; supervision; validation; writing—review and editing. **Nimesh K. Patel**: Conceptualization; investigation; methodology; project administration; supervision; validation; visualization; writing—review and editing.

## TRANSPARENCY STATEMENT

The lead author Prakash Raj Oli affirms that this manuscript is an honest, accurate, and transparent account of the study being reported; that no important aspects of the study have been omitted; and that any discrepancies from the study as planned (and, if relevant, registered) have been explained.

## Supporting information

Supporting information.

Supporting information.

## Data Availability

The data that supports the findings of this study are available in the supplementary material of this article.
