# Supplementary material for: Safety and efficacy of direct oral anticoagulants in comparison to warfarin in obese patients with atrial fibrillation: A systematic review and meta‐analysis
Source: Health Sci Rep. 2024 Apr 21;7(4):e2044. doi: 10.1002/hsr2.2044 (PMC11033493; doi:10.1002/hsr2.2044)
Supplement: Supplementary file 1 — Supporting information. [file HSR2-7-e2044-s001.docx]

**Supplementary files:**

**Safety and efficacy of direct oral anticoagulants in comparison to warfarin in obese patients with atrial fibrillation: A systematic review and meta-analysis**

**Electronic search details**

(((Atrial fibrillation) AND ((DOAC OR 'Direct oral anticoagulants' OR dabigatran OR Pradaxa OR apixaban OR Eliquis OR rivaroxaban OR Xarelto OR edoxaban OR Lixiana))) AND (('Vitamin K antagonist' OR VKA OR Warfarin OR Coumadin OR Jantoven))) AND (Obesity OR Obese)

PubMed

<https://pubmed.ncbi.nlm.nih.gov/?term=%28%28%28Atrial+fibrillation%29+AND+%28%28DOAC+OR+%27Direct+oral+anticoagulants%27+OR+dabigatran+OR+Pradaxa+OR+apixaban+OR+Eliquis+OR+rivaroxaban+OR+Xarelto+OR+edoxaban+OR+Lixiana%29%29%29+AND+%28%28%27Vitamin+K+antagonist%27+OR+VKA+OR+Warfarin+OR+Coumadin+OR+Jantoven%29%29%29+AND+%28Obesity+OR+Obese%29&sort=date&size=200>

82

PubMed central

[https://www.ncbi.nlm.nih.gov/pmc?term=(((Atrial%20fibrillation)%20AND%20((DOAC%20OR%20%27Direct%20oral%20anticoagulants%27%20OR%20dabigatran%20OR%20Pradaxa%20OR%20apixaban%20OR%20Eliquis%20OR%20rivaroxaban%20OR%20Xarelto%20OR%20edoxaban%20OR%20Lixiana)))%20AND%20((%27Vitamin%20K%20antagonist%27%20OR%20VKA%20OR%20Warfarin%20OR%20Coumadin%20OR%20Jantoven)))%20AND%20(Obesity%20OR%20Obese)[all]&cmd=correctspelling](https://www.ncbi.nlm.nih.gov/pmc?term=(((Atrial%20fibrillation)%20AND%20((DOAC%20OR%20%27Direct%20oral%20anticoagulants%27%20OR%20dabigatran%20OR%20Pradaxa%20OR%20apixaban%20OR%20Eliquis%20OR%20rivaroxaban%20OR%20Xarelto%20OR%20edoxaban%20OR%20Lixiana)))%20AND%20((%27Vitamin%20K%20antagonist%27%20OR%20VKA%20OR%20Warfarin%20OR%20Coumadin%20OR%20Jantoven)))%20AND%20(Obesity%20OR%20Obese)%5ball%5d&cmd=correctspelling)

1795

Scopus

<https://www.scopus.com/results/results.uri?sort=plf-f&src=s&st1=%28%28%28Atrial+fibrillation%29+AND+%28%28DOAC+OR+%27Direct+oral+anticoagulants%27+OR+dabigatran+OR+Pradaxa+OR+apixaban+OR+Eliquis+OR+rivaroxaban+OR+Xarelto+OR+edoxaban+OR+Lixiana%29%29%29+AND+%28%28%27Vitamin+K+antagonist%27+OR+VKA+OR+Warfarin+OR+Coumadin+OR+Jantoven%29%29%29+AND+%28Obesity+OR+Obese%29&sid=a397eb775820dd1ef3be289417361a31&sot=b&sdt=b&sl=279&s=TITLE-ABS-KEY%28%28%28%28Atrial+fibrillation%29+AND+%28%28DOAC+OR+%27Direct+oral+anticoagulants%27+OR+dabigatran+OR+Pradaxa+OR+apixaban+OR+Eliquis+OR+rivaroxaban+OR+Xarelto+OR+edoxaban+OR+Lixiana%29%29%29+AND+%28%28%27Vitamin+K+antagonist%27+OR+VKA+OR+Warfarin+OR+Coumadin+OR+Jantoven%29%29%29+AND+%28Obesity+OR+Obese%29%29&origin=searchbasic&editSaveSearch=&yearFrom=Before+1960&yearTo=Present>

34

Embase

<https://www.embase.com/#advancedSearch/resultspage/history.3/page.1/25.items/orderby.date/source>.

333

Cochrane

<https://www.cochranelibrary.com/advanced-search>

15

Table 1. JBI critical appraisal checklist for observational studies

| JBI critical appraisal checklist for observational studies | Alberts et al. | Berger, et. al | Boivin-Proulx, et. al | Briasoulis, et. al | Costa, et. al | Deitelzweig, et al., 2020 | Deitelzweig, et al., 2022 | Huang, et al. | Kido, et al. | Kushnir, et al. | Nakao, et al. | Patil, et al. | Perales, et al. | Peterson, et al. | Russo, et al. | Weir, et al. |
| --- | --- | --- | --- | --- | --- | --- | --- | --- | --- | --- | --- | --- | --- | --- | --- | --- |
| Were the two groups similar and recruited from the same population? | Yes | Yes | Yes | Yes | Yes | Yes | Yes | Yes | Yes | Yes | Yes | Yes | Yes | Yes | Yes | Yes |
| Were the exposures measured similarly to assign people to both exposed and unexposed groups? | Yes | Yes | Yes | Yes | Yes | Yes | Yes | Yes | Yes | Yes | Yes | Yes | Yes | Yes | Yes | Yes |
| Was the exposure measured in a valid and reliable way? | Yes | Yes | Yes | Yes | Yes | Yes | Yes | Yes | Yes | Yes | Yes | Yes | Yes | Yes | Yes | Yes |
| Were confounding factors identified? | Yes | Yes | Yes | Yes | Yes | Yes | Yes | Yes | Yes | Yes | Yes | No | No | Yes | Unclear | Yes |
| Were strategies to deal with confounding factors stated? | Yes | Yes | Yes | Yes | Yes | Yes | Yes | Yes | Yes | Yes | Unclear | No | No | Yes | Unclear | Yes |
| Were the groups/participants free of the outcome at the start of the study (or at the moment of exposure)? | Yes | Yes | Yes | Yes | Yes | Yes | Yes | Yes | Yes | Yes | Yes | Yes | Yes | Yes | Yes | Yes |
| Were the outcomes measured in a valid and reliable way? | Yes | Yes | Yes | Yes | Yes | Yes | Yes | Yes | Yes | Yes | Yes | Yes | Yes | Yes | Yes | Yes |
| Was the follow-up time reported and sufficient to be long enough for outcomes to occur? | Yes | Yes | Yes | Yes | Yes | Yes | Yes | Yes | Yes | Yes | Yes | Yes | Yes | Yes | Yes | Yes |
| Was follow up complete, and if not, were the reasons to loss to follow up described and explored? | Unclear | Yes | Yes | Yes | Yes | Yes | Yes | Yes | Yes | Yes | Yes | Yes | No | Yes |  | Yes |
| Were strategies to address incomplete follow up utilized? | Unclear | Yes | Yes | Yes | Yes |  | Unclear | Unclear | Unclear | Yes | Unclear | Unclear | Unclear | Unclear |  | Unclear |
| Was an appropriate statistical analysis used? | Yes | Yes | Yes | Yes | Yes | Yes | Yes | Yes | Yes | Yes | Yes | Yes | Yes | Yes | Yes | Yes |
| Study population site | IBM MarketScan Commercial Claims and Encounters (CCAE) and IBM MarketScan Medicare Supplemental (MDCR) | The IQVIA PharMetricsi Plus data | RAMQ and Med-Echo databases | Veterans Health Administration (VHA) Corporate Data Warehouse (CDW) | US Optum deIdentified Electronic Health Record data | (FFS) Medicare data from the US Centers for Medicare & Medicaid Services (CMS) and four US commercial claims databases: the IBM MarketScan® Commercial Claims and Encounter Database, the IQVIA PharMetrics Plus™ Database, the Optum Clinformatics™ Data Mart, and the Humana Research Database. | Medicare data from the United States Centers for Medicare and Medicaid Serv- ices database and from the Veterans Affairs database | Kaiser Permanente SouthernCalifornia (KPSC) | a tertiary care hospital in a Midwestern state. | Montefiore Medical Center (Bronx, NY, USA) | UK | Salem Veterans Affairs Medical Center (SVAMC) | Southern Arizona | Truven MarketScan Commercial Claims and Encounters and Medicare Supplemental databases f | Monaldi Hospital, Naples, and University of Campania “Luigi Vanvitelli,” Naples | US |
| Overall appraisal | Include | Include | Include | Include | Include | Include | Include | Include | Include | Include | Include | Include | Include | Include | Include | Include |

**
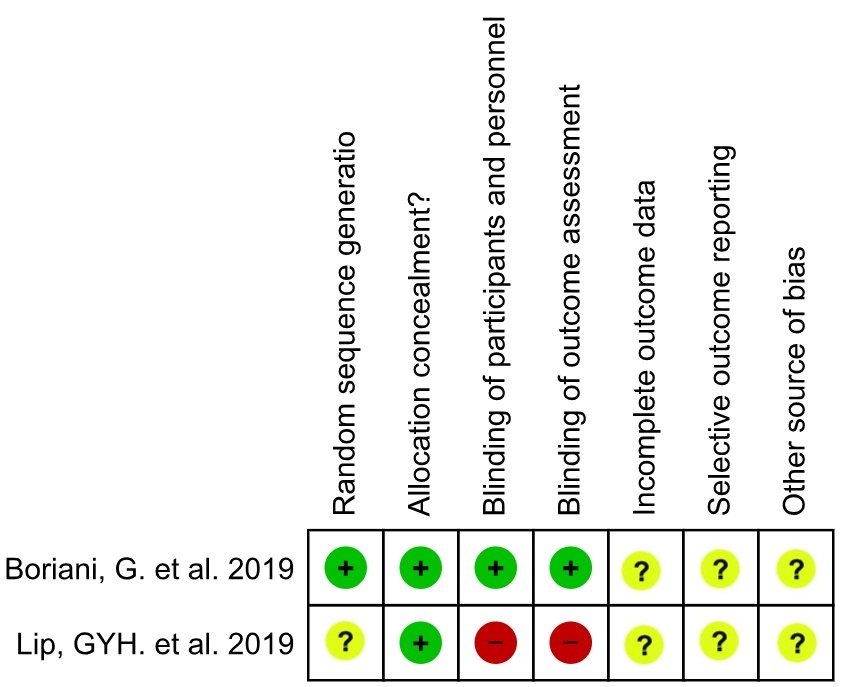
**

**eFigure 1. Bias summary of RCTs**

**Table 2.** Baseline clinical characteristics and concomitant drugs used among included participants.

| **Study** | **Groups** | **Baseline clinical characteristics** | | | | **Baseline concomitant drug use, %** | | | |
| --- | --- | --- | --- | --- | --- | --- | --- | --- | --- |
|  |  | QCI score [mean (SD)] | CHA_2_DS_2_-  VASc score  [mean (SD)] | Charlson score | HAS-BLED score [mean (SD)] | Non-oral anticoagulant | Antihyperlipidemic | Antihypertensives | Antiplatelet agents |
| Alberts, MJ. et al. ^11^ | Rivaroxaban | 1.51 (1.94) | 2.64 (1.79) |  | 2.18(1.37) | 11.3 | 7.5 | 91.2 | 11.0 |
|  | Warfarin | 2.03 (2.12) | 3.42 (1.91) |  | 2.44(1.47) | 12.7 | 12.3 | 94.7 | 10.3 |
| Berger, JS. et al. ^12^ | Rivaroxaban | 1.79 ± 2.18 | 2.65 ± 1.59 |  | 1.80 ± 1.12 | 16.7 | 50.2 | 50.2 | 10.5 |
|  | Warfarin | 1.75 ± 1.92 | 2.70 ± 1.54 |  | 1.79 ± 1.08 | 16.4 | 50.8 | 50.8 | 9.1 |
| Boivin-Proulx, LA. et al. ^13^ | Rivaroxaban |  | 2.60 (1.28) | 5.11 (3.35) | 3.35 (1.43) |  | 57.31 |  | 43.18 |
|  | Apixaban |  | 2.71 (1.22) | 5.15 (3.49) | 3.50 (1.48) |  | 54.25 |  | 38.22 |
|  | Warfarin |  | 2.60 (1.28) | 4.92 (3.30) | 3.35 (1.44) |  | 53.52 |  | 41.18 |
| Boriani, G. et al. ^14^ | Higher dose edoxaban |  | 2.86 (0.96) |  |  |  |  |  | 29.73 |
|  | Lower dose edoxaban |  | 2.79 (0.76) |  |  |  |  |  | 28.96 |
|  | Warfarin |  | 2.65 (0.81) |  |  |  |  |  | 31.24 |
| Briasoulis A. et al. ^15^ | _Apixaban_ |  |  |  |  |  | 62.4 |  |  |
|  | _Dabigatran_ |  |  |  |  |  | 60.6 |  |  |
|  | _Rivaroxaban_ |  |  |  |  |  | 59.7 |  |  |
|  | _Warfarin_ |  |  |  |  |  | 65.2 |  |  |
| Costa, OS. et al. ^16^ | _Rivaroxaban_ |  | 3 (2, 4) | 2 (1, 3) |  |  | 63.8 |  | 55.8 |
|  | _Warfarin_ |  | 3 (2, 4) | 2 (1, 3) |  |  | 65.6 |  | 54.7 |
| Deitelzweig, S. et al. 2020 ^17^ | Apixaban |  | 3.9 (1.7) | 3.2 1.4 | 3.7 (2.9) |  | 63.9 |  | 21.6 |
|  | Dabigatran |  | 3.7 (1.7) | 3.0 1.4 | 3.3 (2.8) |  | 60.3 |  | 17.3 |
|  | Rivaroxaban |  | 3.7 (1.7) | 3.1 1.4 | 3.4 (2.8) |  | 61.5 |  | 18.8 |
|  | Warfarin |  | 4.3 (1.6) | 3.5 1.4 | 4.5 (3.1) |  | 65.7 |  | 22.2 |
| Deitelzweig S et al 2022 ^18^ | Apixaban |  | 3.8 (1.5) | 2.6 (2.4) | 2.6 (1.1) |  |  |  |  |
|  | Warfarin |  | 3.8 (1.6) | 3.1 (2.6) | 2.7 (1.1) |  |  |  |  |
| Huang, CW. et al. ^19^ | Dabigatran |  | 3 (2, 4) | 2 (1, 4) | 2 (1, 3) |  | 70.9 |  | 31.1 |
|  | Warfarin |  | 3 (2, 5) | 2 (1, 4) | 2 (1, 3) |  | 70.9 |  | 29.6 |
| Kido, K. et.al ^20^ | _DOAC_ |  |  |  |  |  |  |  | 53.13 |
|  | _Warfarin_ |  |  |  |  |  |  |  | 50.01 |
| Kushnir M et al ^21^ | Apixaban |  | 3·5 (1·6) | 0·0  (0·0–2·0) |  |  |  |  |  |
|  | Rivaroxaban |  | 3·1 (1·5) | 1·0  (0·0–2·0) |  |  |  |  |  |
|  | Warfarin |  | 4.1 (1·8) | 2.0  (1·0–4·0) |  |  |  |  |  |
| Lip, GYH. et al. ^22^ | Edoxaban |  | 2.7 (1.4) |  | 0.8 (0.8) |  | 41.0 |  | 17.4 |
|  | Warfarin |  | 2.7 (1.4) |  | 0.9 (0.8) |  | 40.9 |  | 21.2 |
| Nakao, YM. et al ^23^ | _DOAC_ |  |  |  |  |  | 60.90 |  | 50.52 |
|  | _Warfarin_ |  |  |  |  |  | 60.65 |  | 33.74 |
| Patil, T. et al. ^24^ | _DOAC_ |  | 3.59 ± 1.24 |  | 2.41 ± 1.06 |  |  |  | 50.39 |
|  | _Warfarin_ |  | 3.48 ± 1.19 |  | 2.29 ± 0.95 |  |  |  | 54.21 |
| Perales, IJ. et al. ^25^ | Rivaroxaban |  |  |  |  |  |  |  |  |
|  | Warfarin |  |  |  |  |  |  |  |  |
| Peterson, ED. et al. ^26^ | Rivaroxaban | 1.80 (2.00) | 3.21 (1.79) |  | 2.25 (1.38) |  |  |  |  |
|  | Warfarin | 2.60 (2.34) | 3.85 (1.92) |  | 2.75 (1.57) |  |  |  |  |
| Russo, V. et al. ^27^ | _DOAC_ |  | 2.5 (1.8) |  | 2.9 (2.2) |  |  |  | 34.8 |
|  | _Warfarin_ |  | 3.4 (1.2) |  | 3.6 (1.1) |  |  |  | 34.9 |
| Weir, MR. et al. ^28^ | Rivaroxaban | 2.59 (2.24) | 4.27 (1.69) |  | 3.18 (1.44) | 14.2 | 72.4 | 92.7 | 15.3 |
|  | Warfarin | 3.23 (2.31) | 4.79 (1.69) |  | 3.49 (1.50) | 16.6 | 70.2 | 90.8 | 17.3 |
|  |  |  |  |  |  |  |  |  |  |


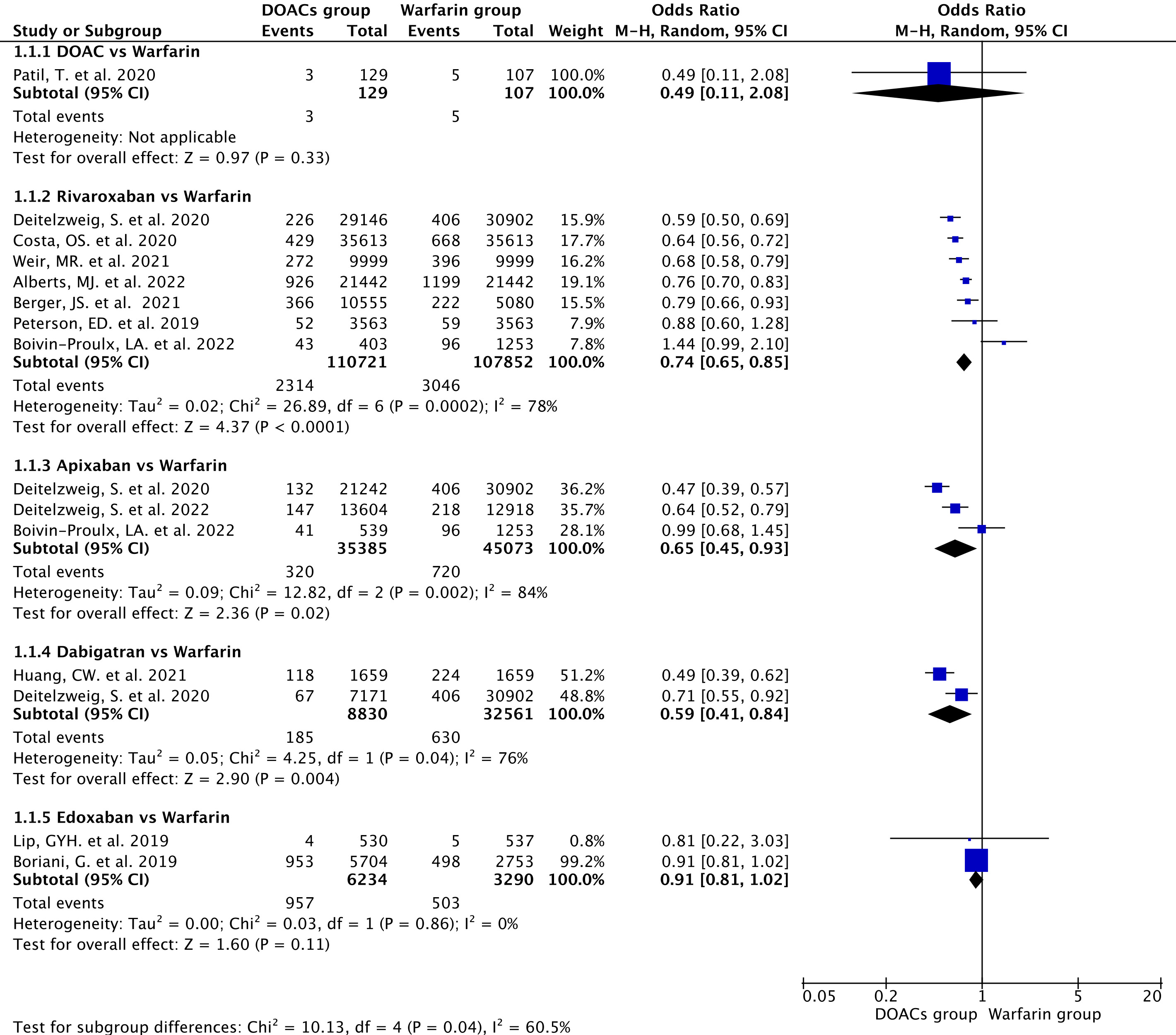


eFigure 2. Forest plot showing a significantly lower occurrence of composite events in the rivaroxaban, apixaban, and dabigatran sub-groups but not in the edoxaban sub-group in comparison to the Warfarin group using the random effect model.

**
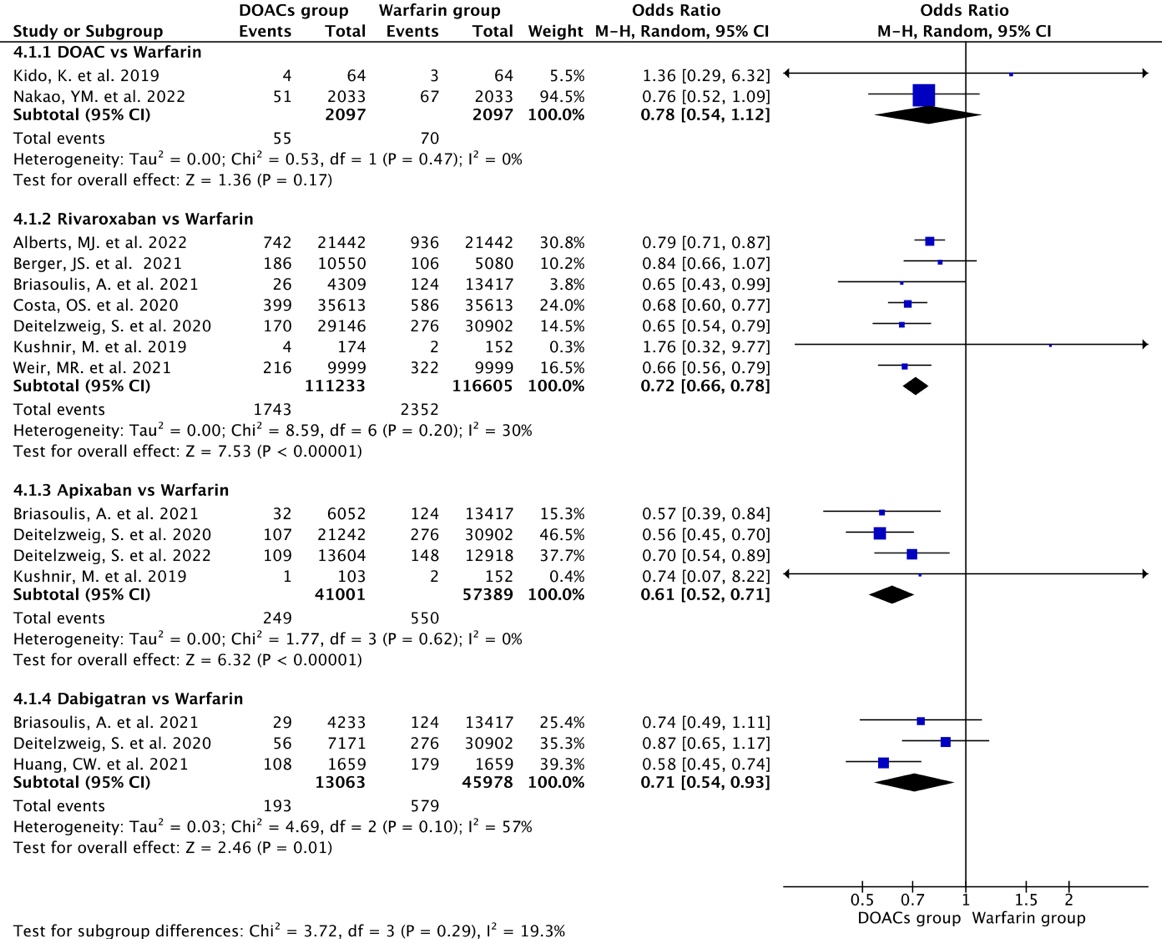
**

3(a)


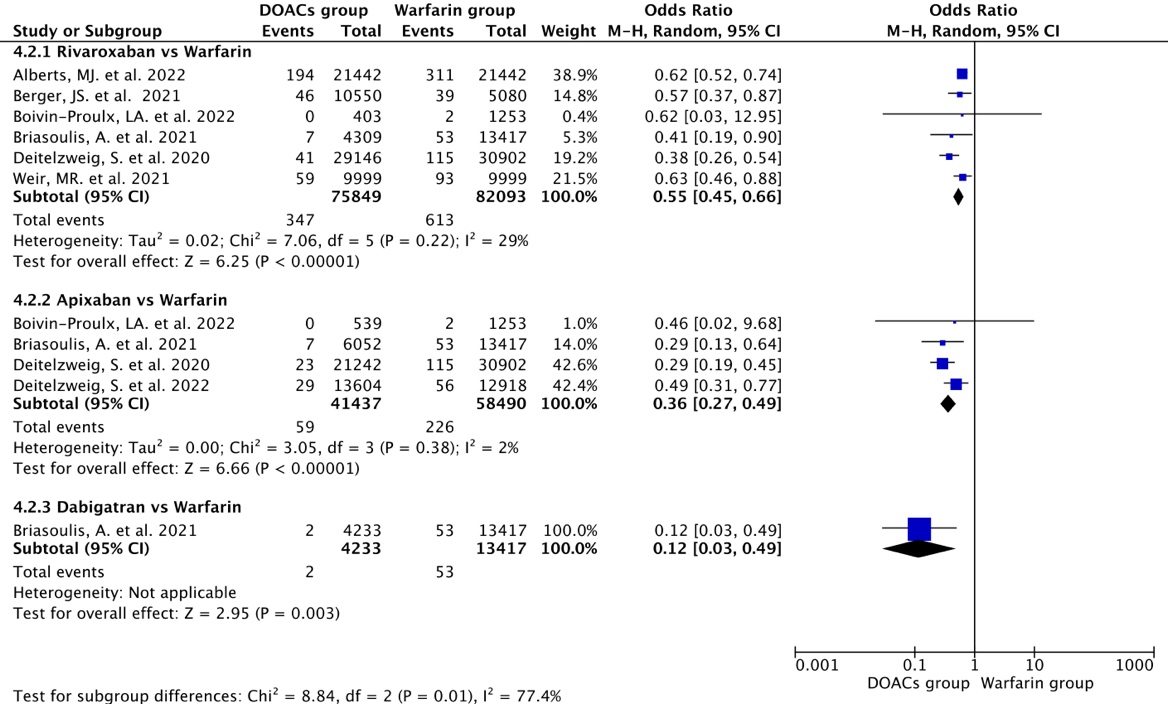


4(b)

3(b)

eFigure 3(a) and 3(b). Forest plot showing significantly lower occurrence of stroke events in the rivaroxaban, apixaban, and dabigatran sub-groups in comparison to the Warfarin group using the random effect model: (a) for ischemic stroke, and (b) for hemorrhagic stroke.


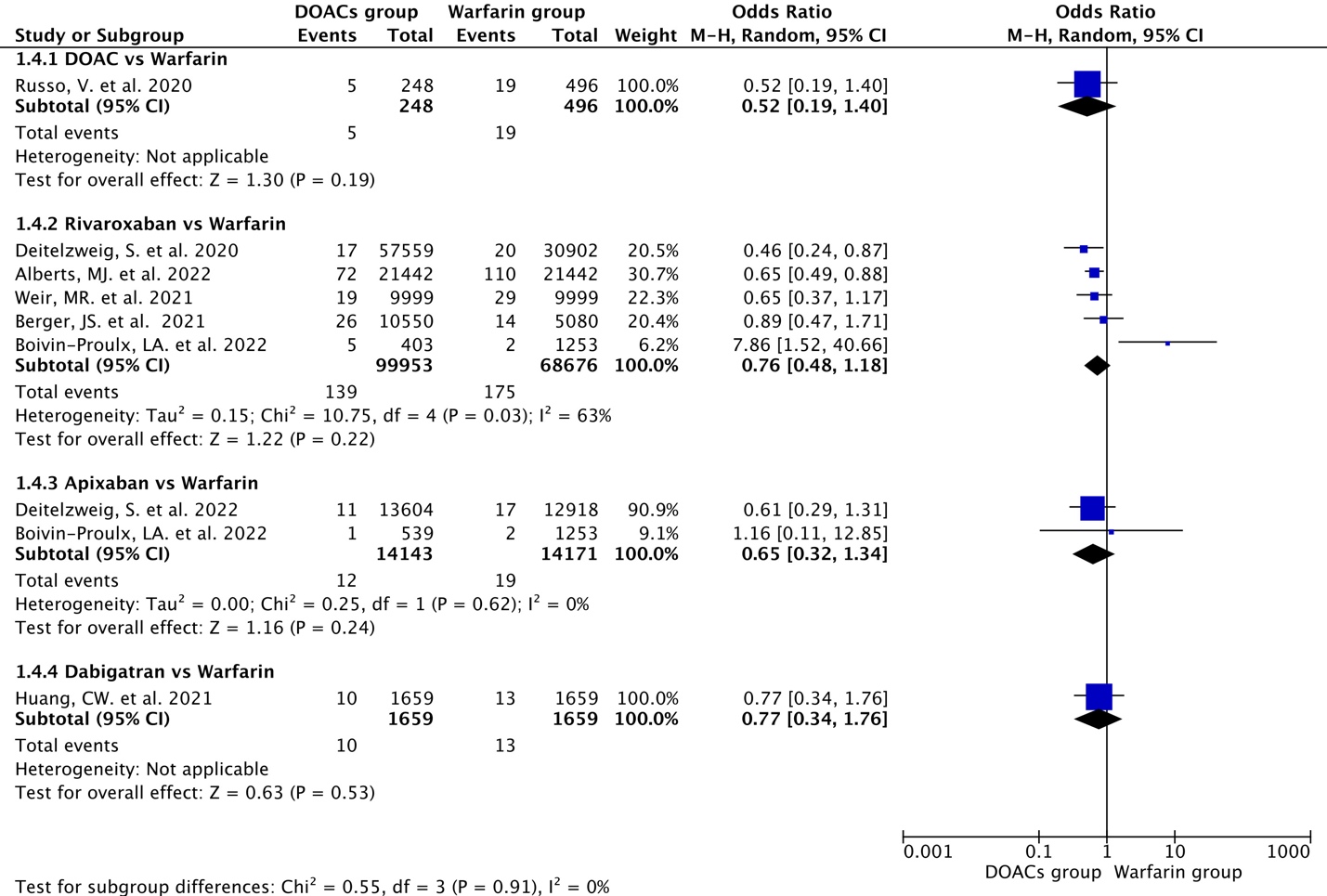


eFigure 4. Forest plot showing no significantly lower occurrence of systemic embolic events in the rivaroxaban, apixaban, and dabigatran sub-groups in comparison to the Warfarin group using the random effect model.


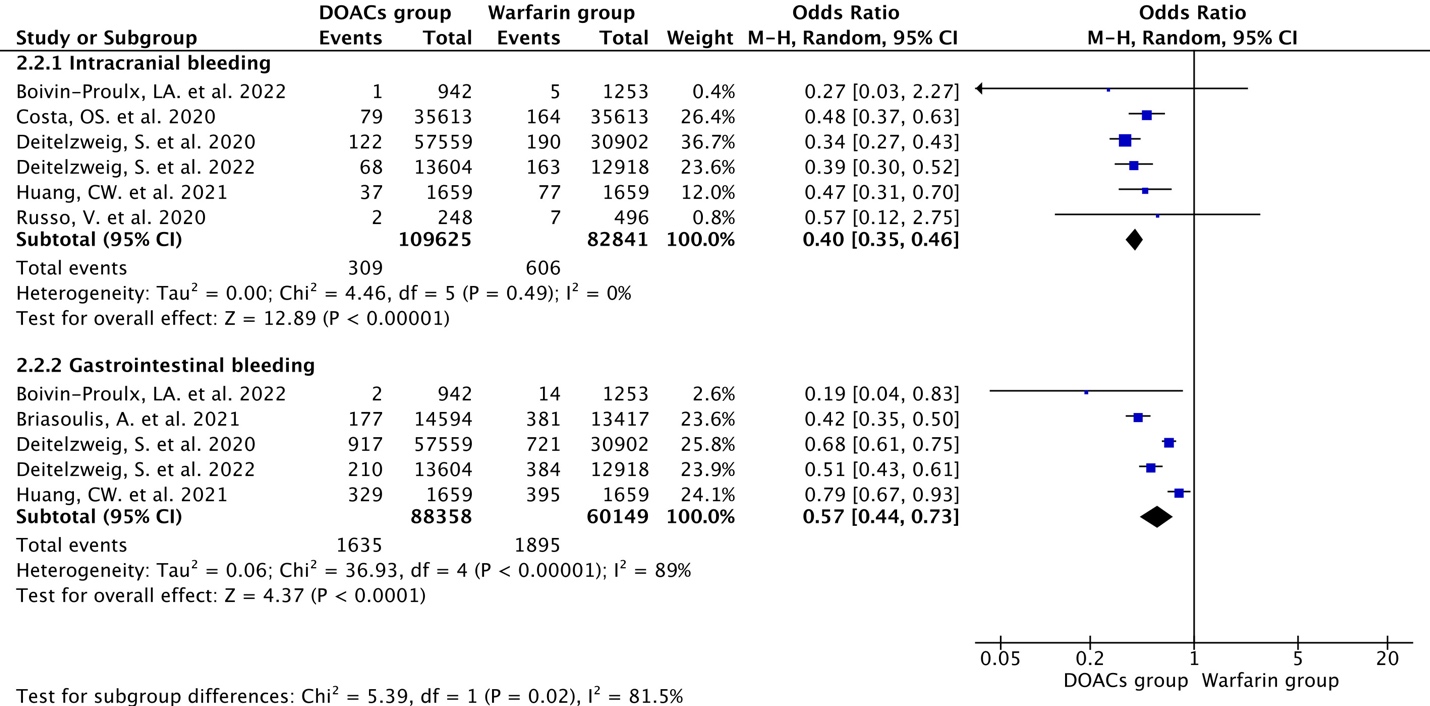
eFigure 5. Forest plot showing significantly lower occurrence of ICB and GIB events in the DOAC group in comparison to the Warfarin group using the random effect model.


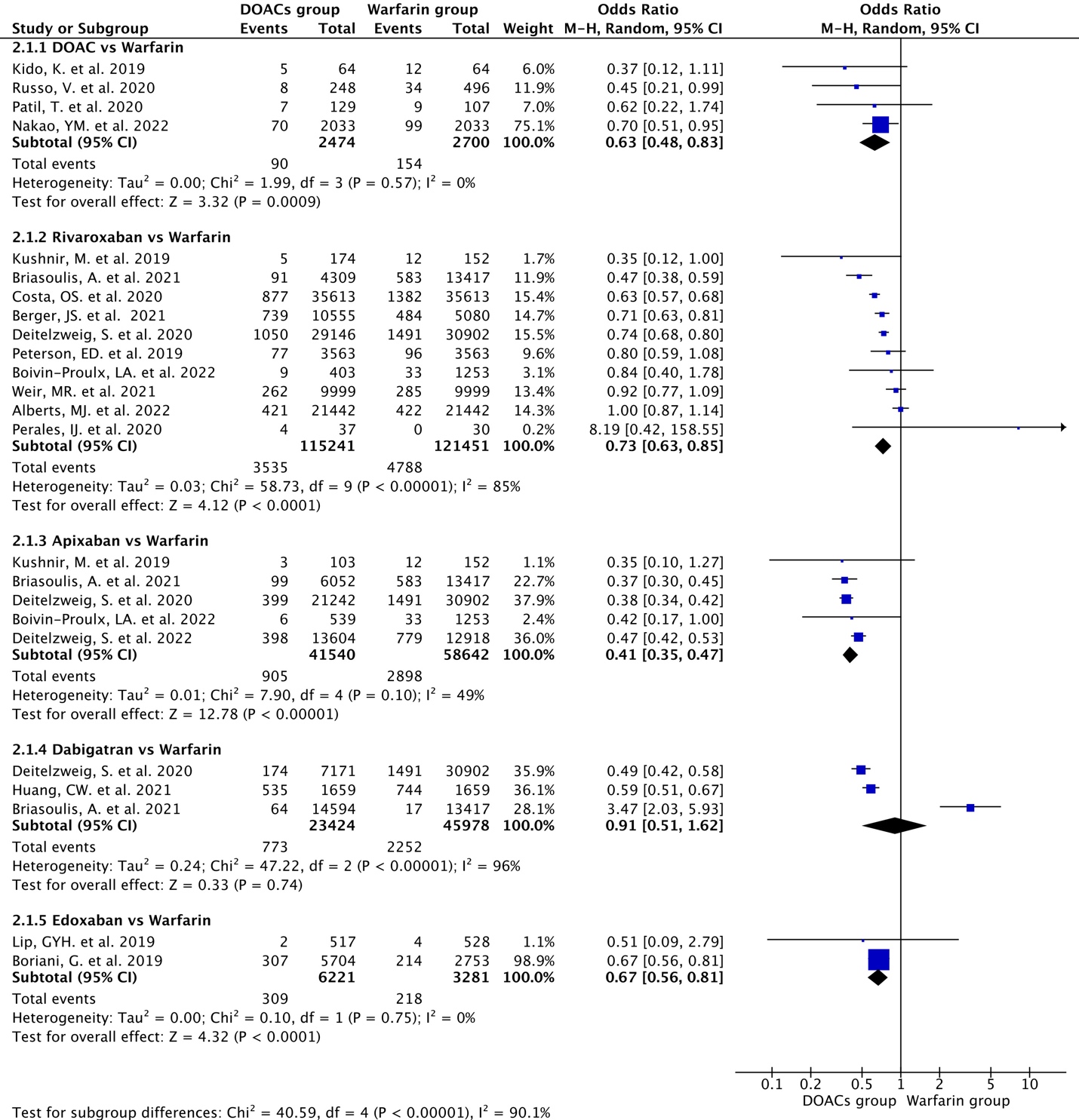
eFigure 6. Forest plot showing the significantly lower occurrence of major bleedings in the rivaroxaban, apixaban, and edoxaban sub-groups, but not in the dabigatran sub-group, in comparison to the Warfarin group using the random effect model.


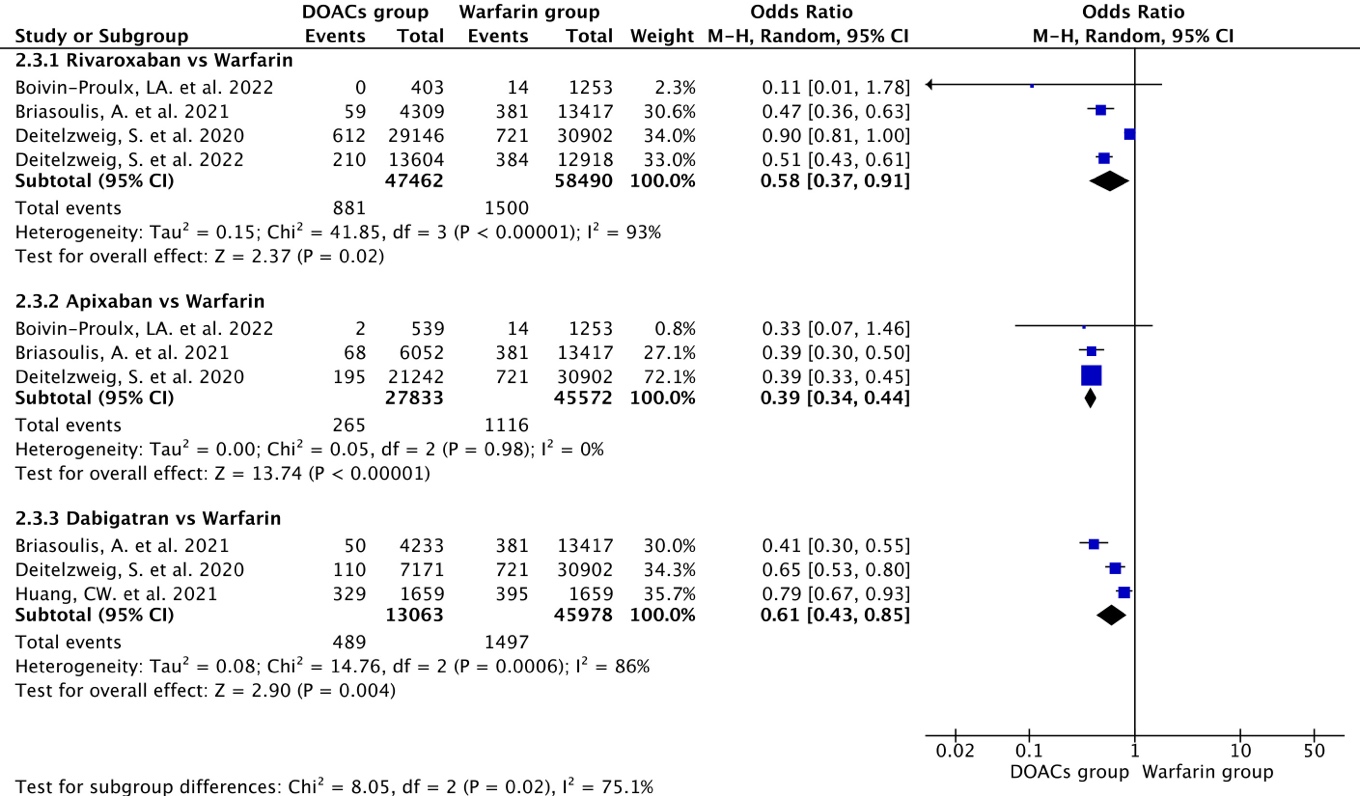


eFigure 7. Forest plot showing significantly lower occurrence of GI bleedings in the rivaroxaban, apixaban, and dabigatran sub-groups in comparison to the Warfarin group using random effect model.


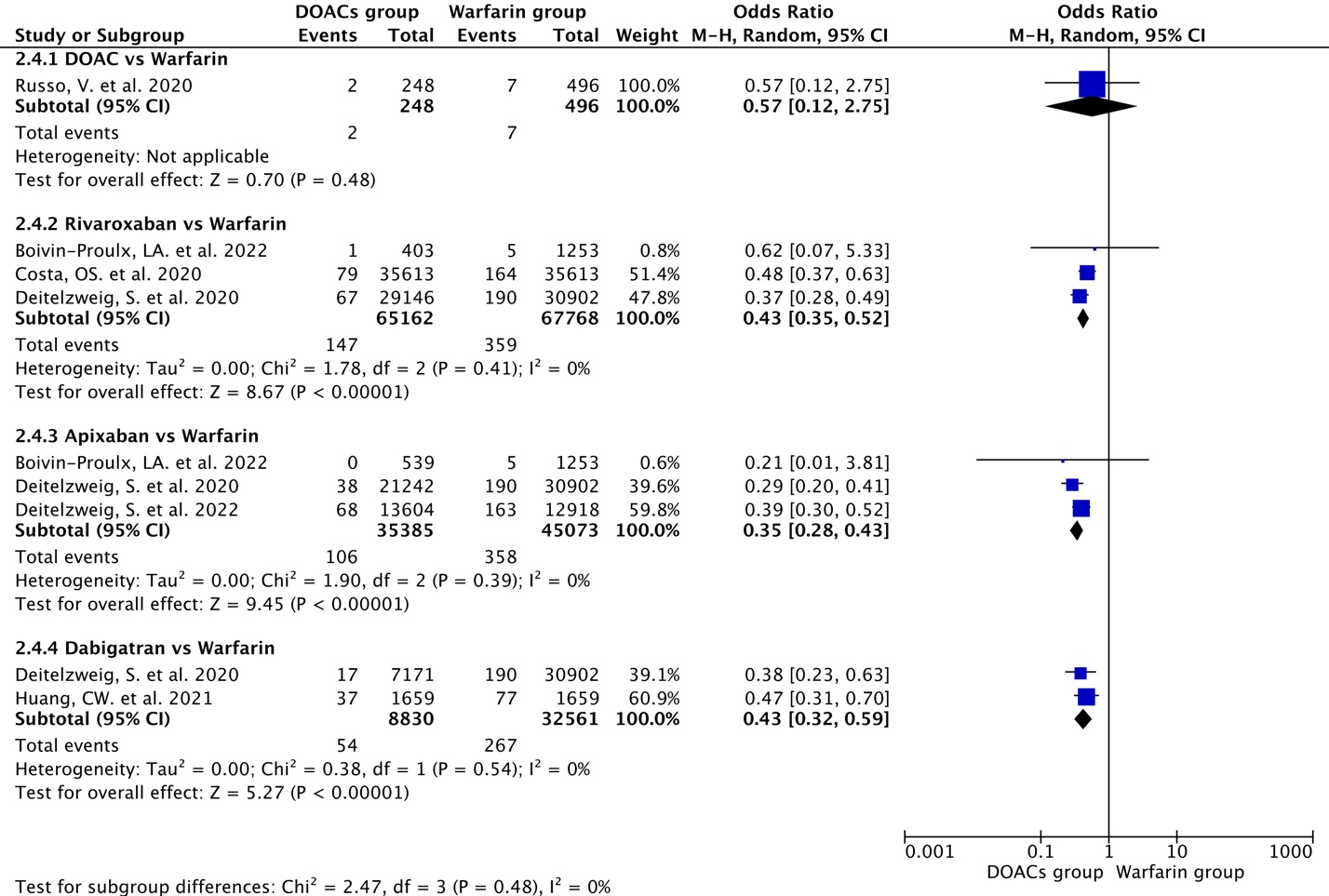


eFigure 8. Forest plot showing significantly lower occurrence of intracranial bleeding in the rivaroxaban, apixaban, and dabigatran sub-group in comparison to the Warfarin group using the random effect model.


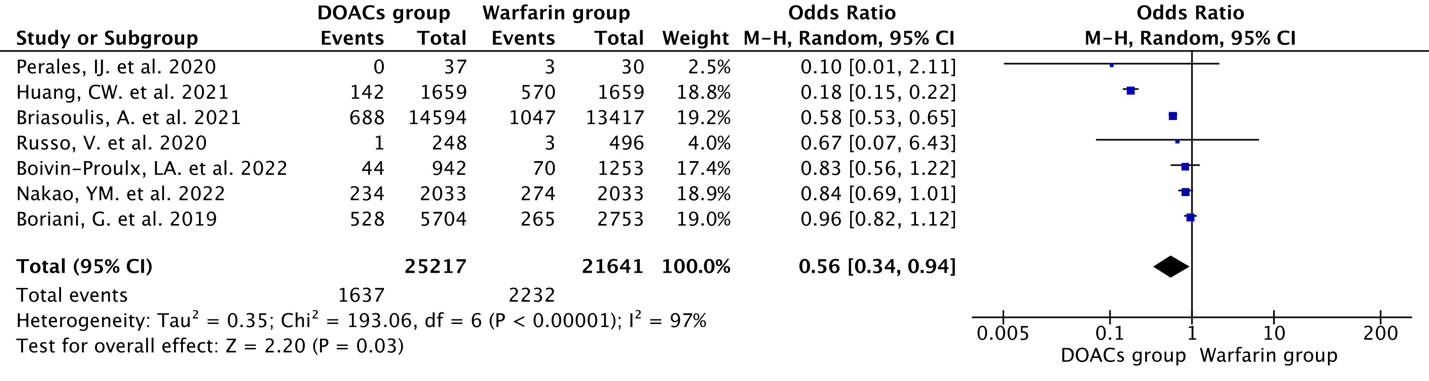


eFigure 9. Forest plot showing significantly lower mortality in the DOAC group in comparison to the Warfarin group using the random effect model.

**
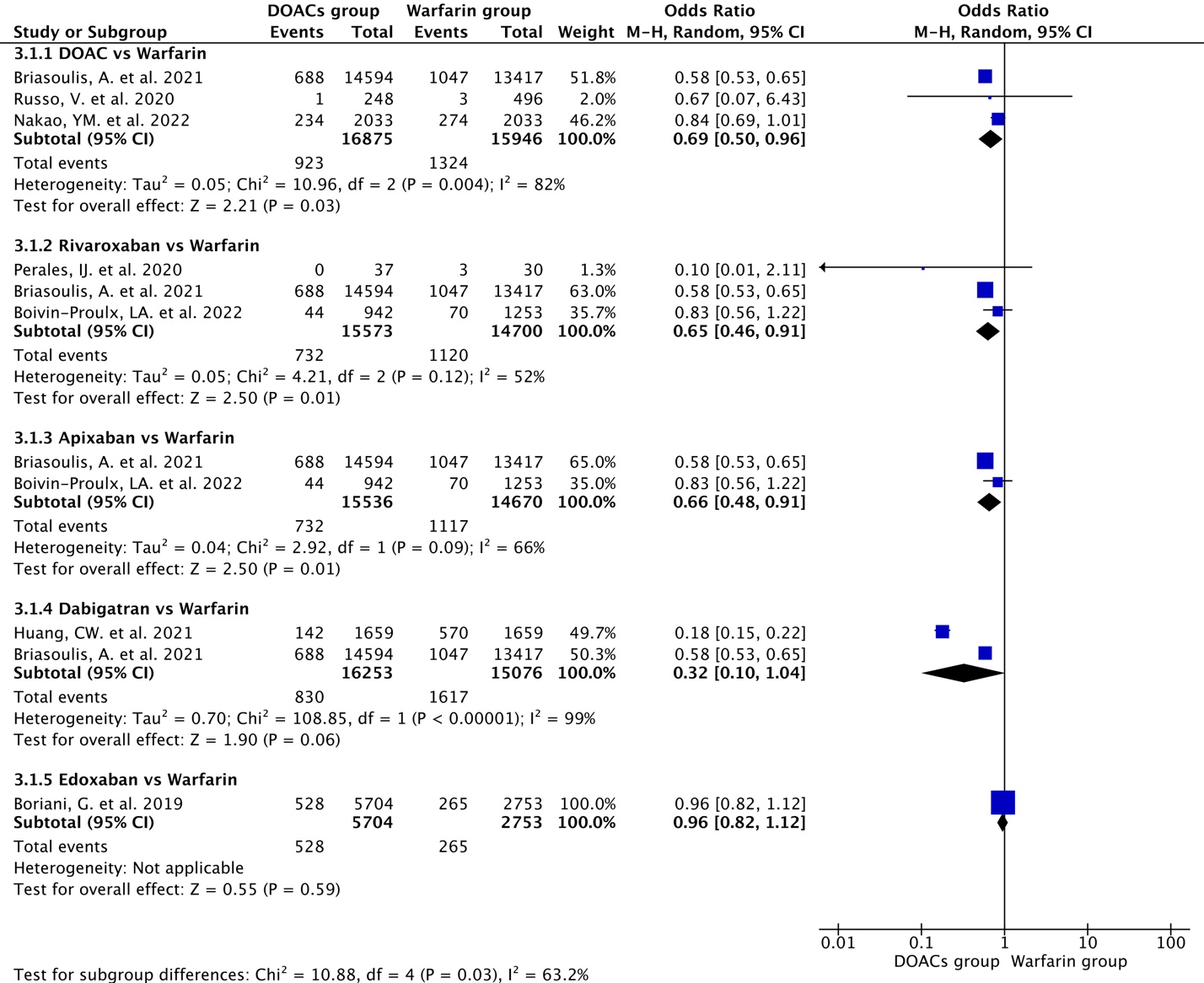
**

**eFigure 10.** Forest plot showing significantly reduced all-cause mortality in the rivaroxaban, and apixaban sub-groups but not in dabigatran and edoxaban sub-groups in comparison to the Warfarin group using the random effect model.

**
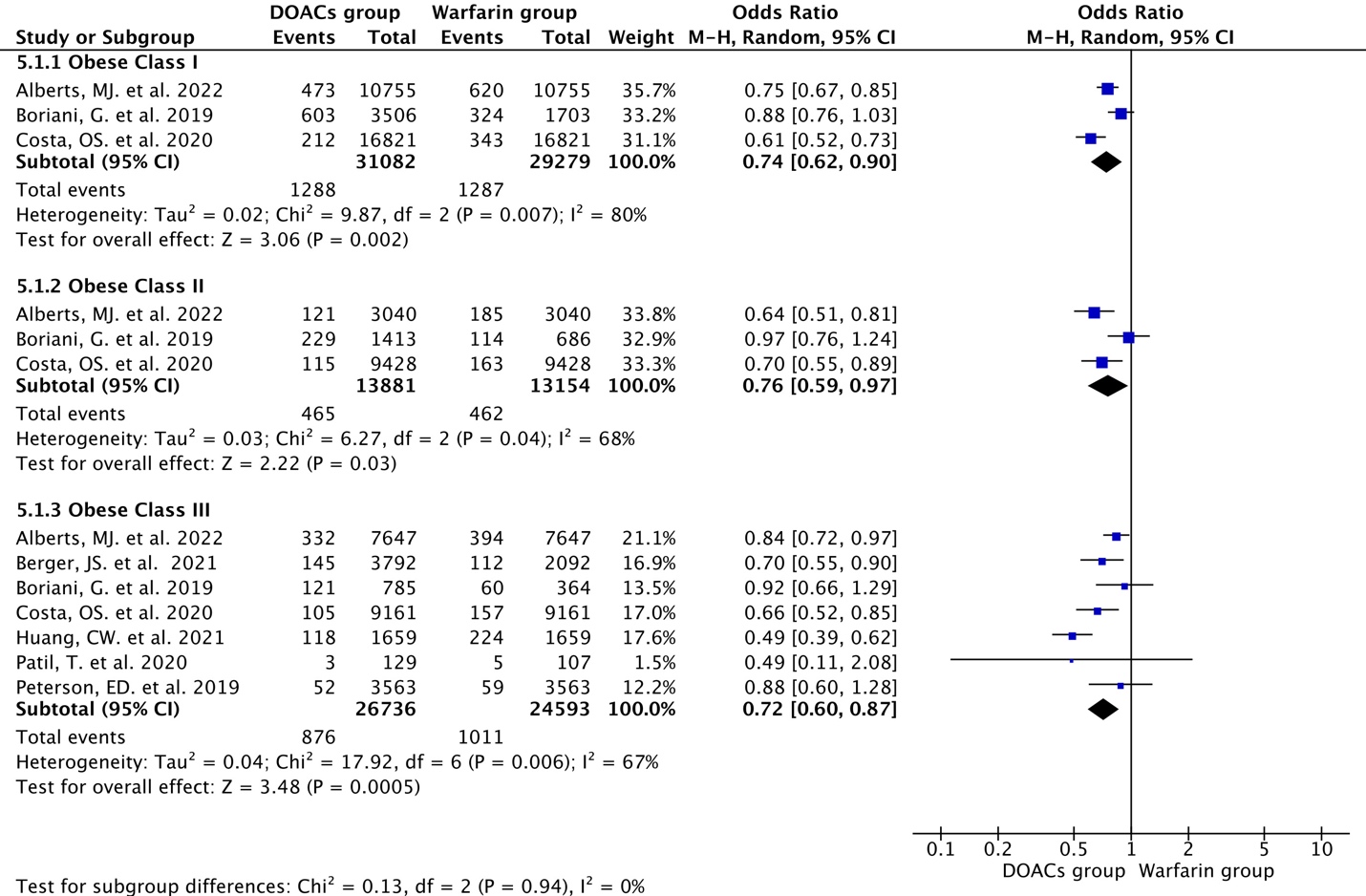
eFigure 11.** Forest plot showing significantly lower occurrence of composite outcome in the obesity class I, obesity class II, and obesity class III in comparison to the Warfarin group using the random effect model.


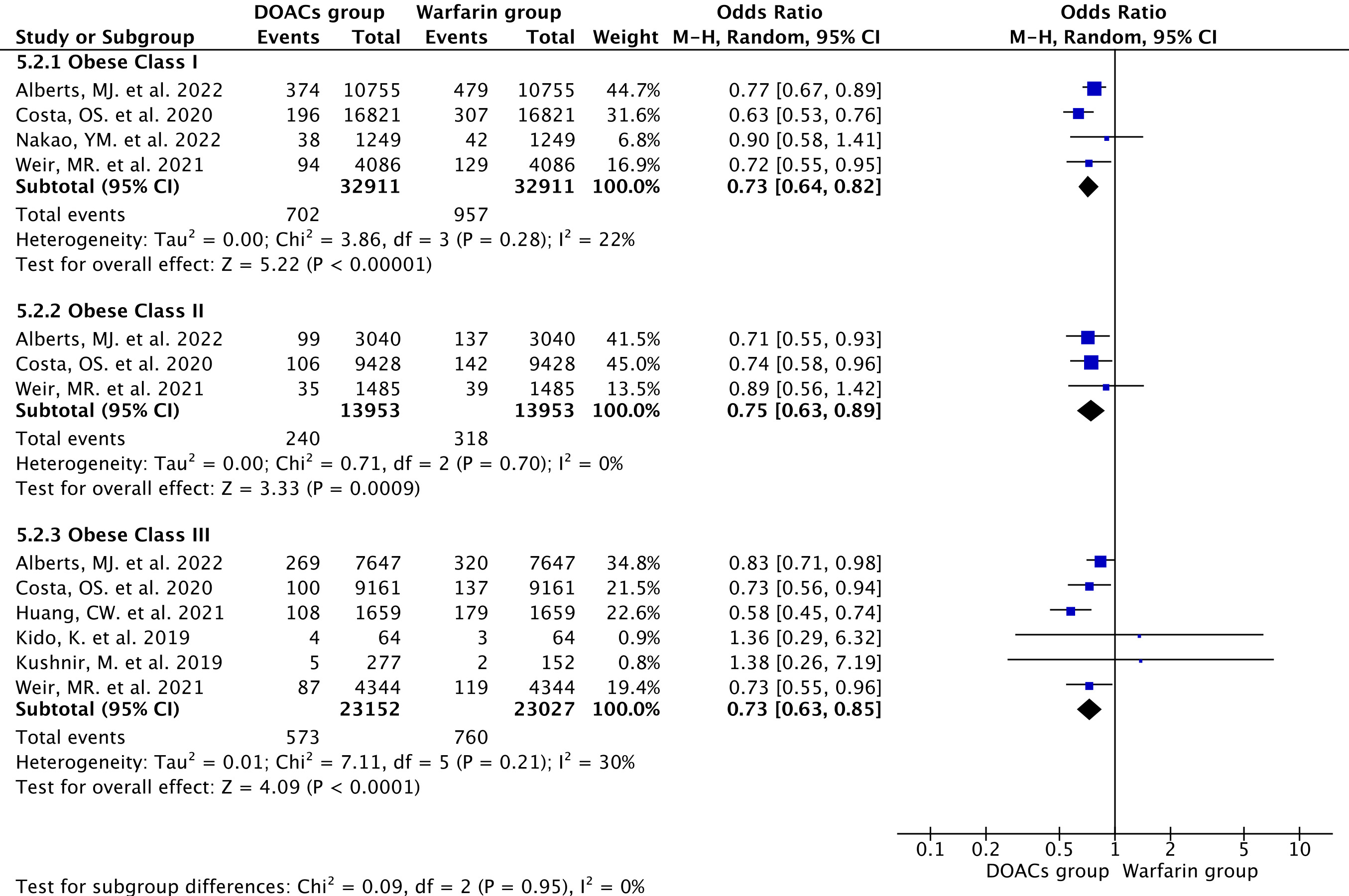


**eFigure 12.** Forest plot showing a significantly lower occurrence of ischemic stroke in the obesity class I, II, and III in comparison to the Warfarin group using the random effect model.

**
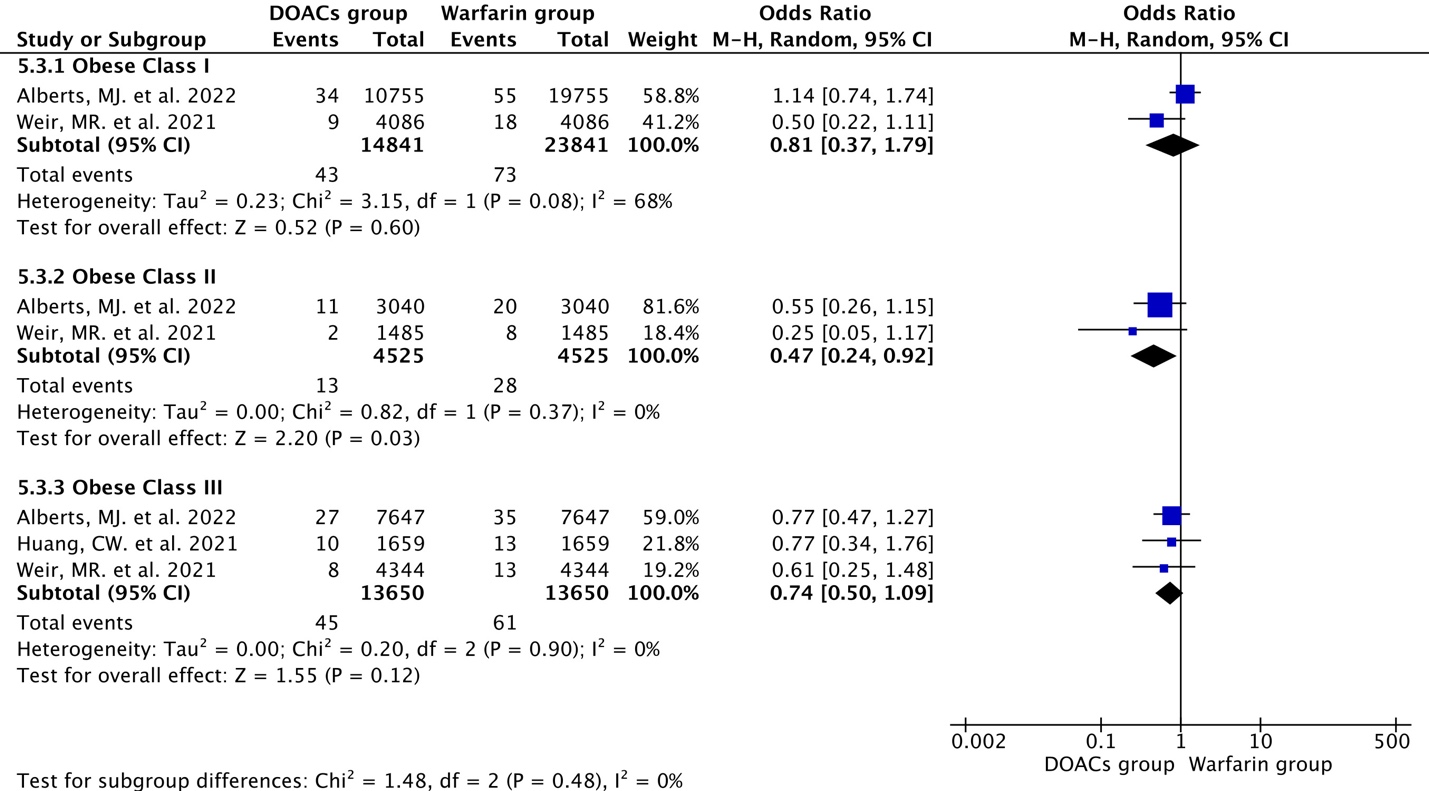
**

**eFigure 13.** Forest plot showing a significantly lower occurrence of the systemic embolic event only in the obesity class II, but not in obesity class I or class III, in comparison to the Warfarin, group using the random effect model.


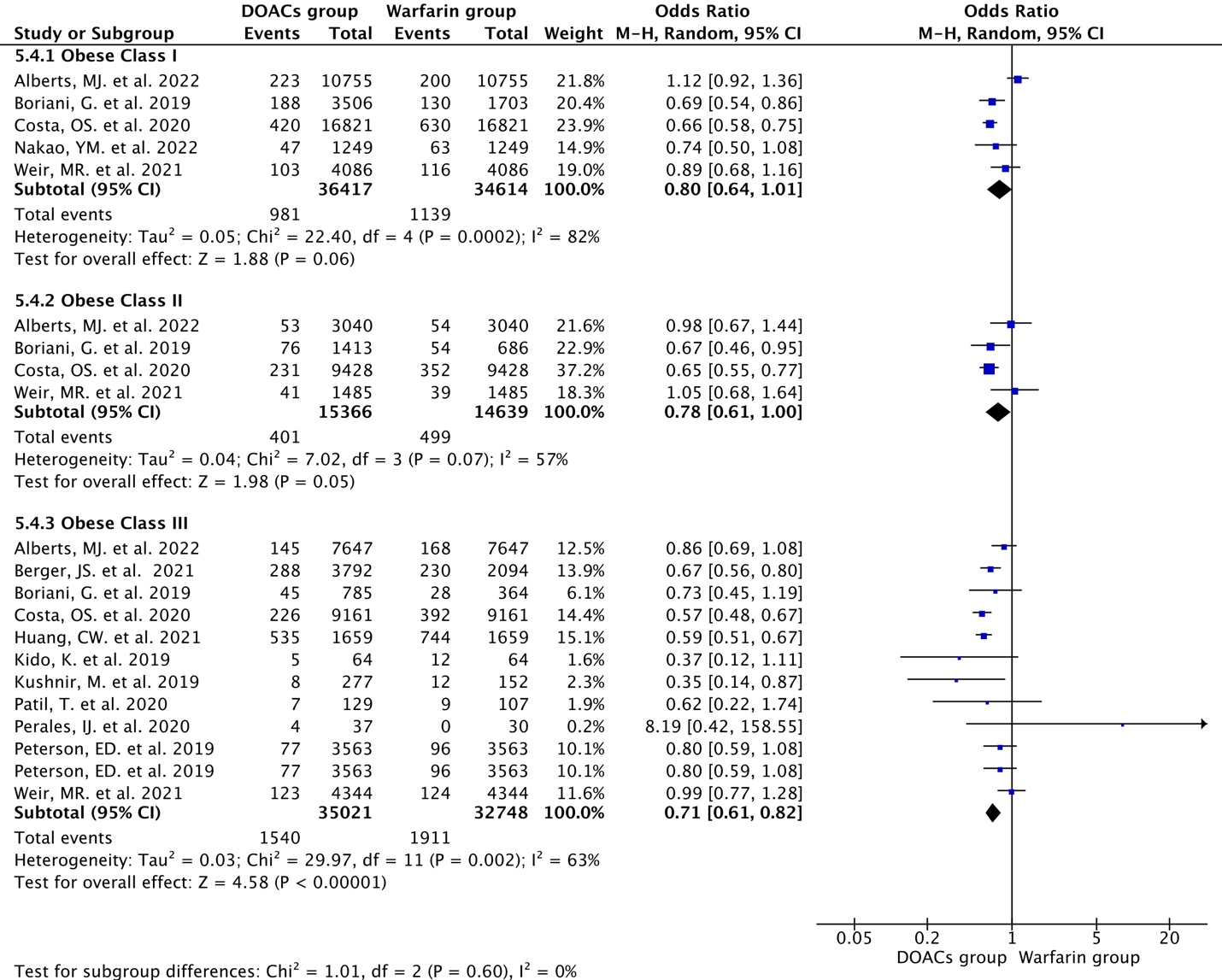


**eFigure 14.** Forest plot showing significantly lower occurrence of major bleeding in the obesity class III but not in class I or class II in comparison to the Warfarin group using the random effect model.


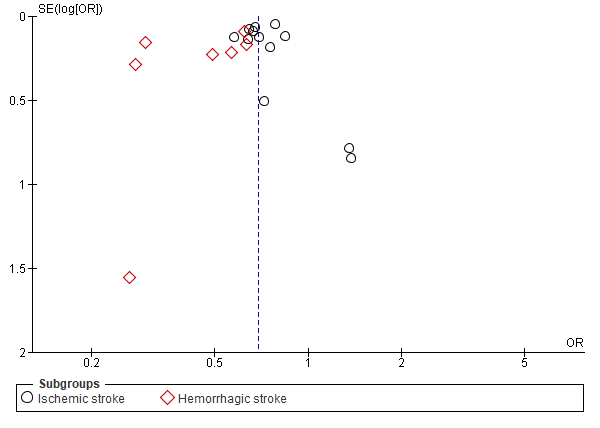

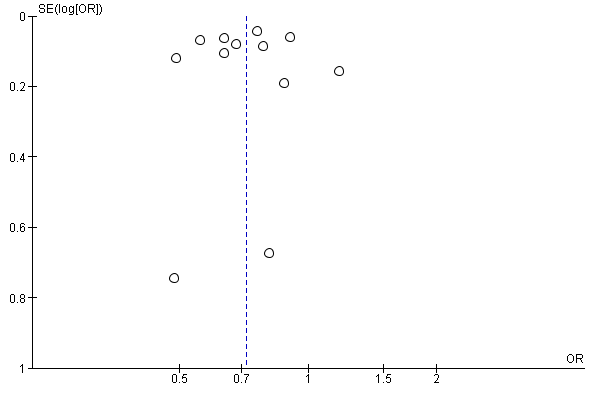


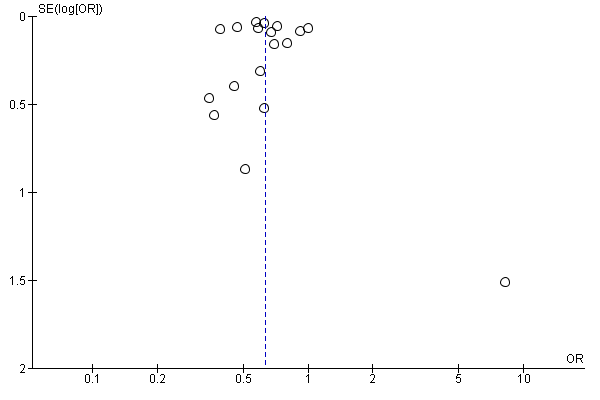


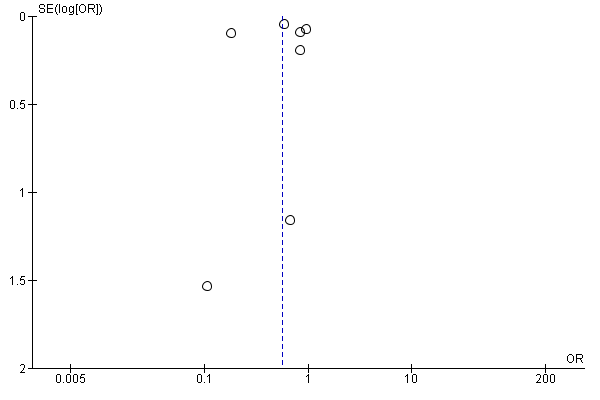


eFigure 15. Publication bias for the composite outcome, stroke, all-cause mortality, and major bleeding
